# Supplementary figures and images for: Variability in performance of genetic-enhanced DXA-BMD prediction models across diverse ethnic and geographic populations: A risk prediction study
Source: PLoS Med. 2024 Aug 30;21(8):e1004451. doi: 10.1371/journal.pmed.1004451 (PMC11404845; doi:10.1371/journal.pmed.1004451)

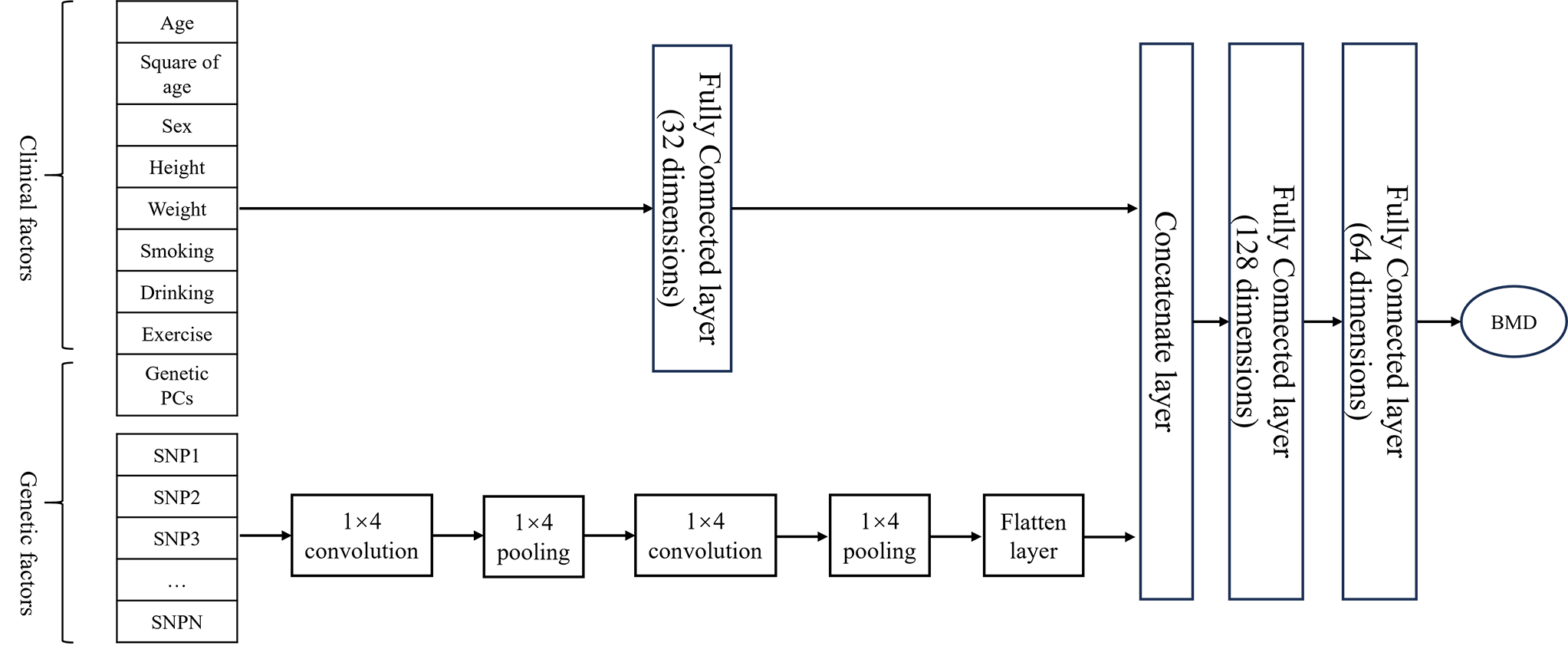

Supplement: S1 Fig — The convolutional layers are 1-dimensional (1 × 4) with pooling layers. (TIF) [file pmed.1004451.s007.tif]

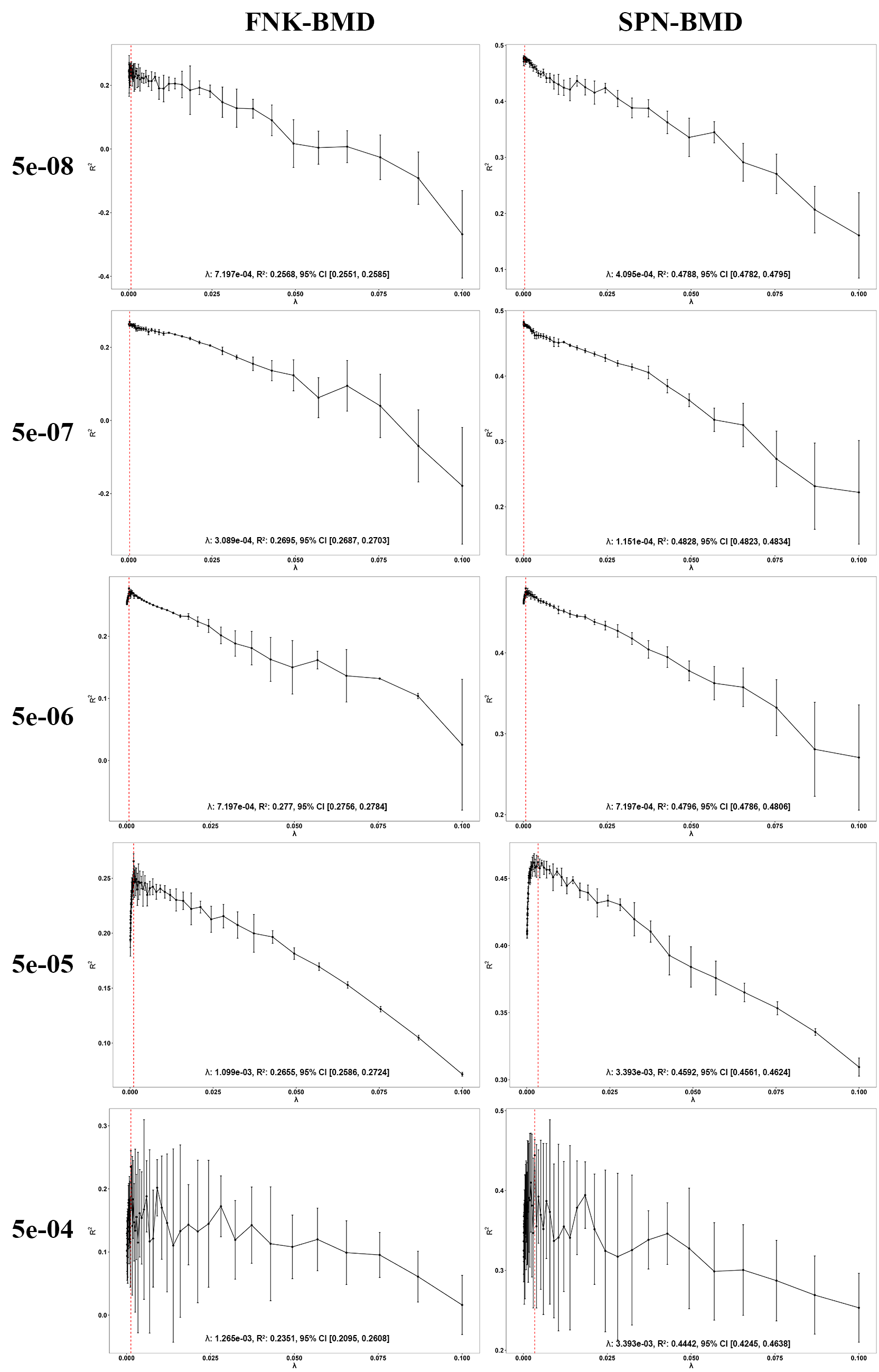

Supplement: S2 Fig — The R2 (coefficient of determination) were calculated within the UKBB Model Selection set. (TIF) [file pmed.1004451.s008.tif]

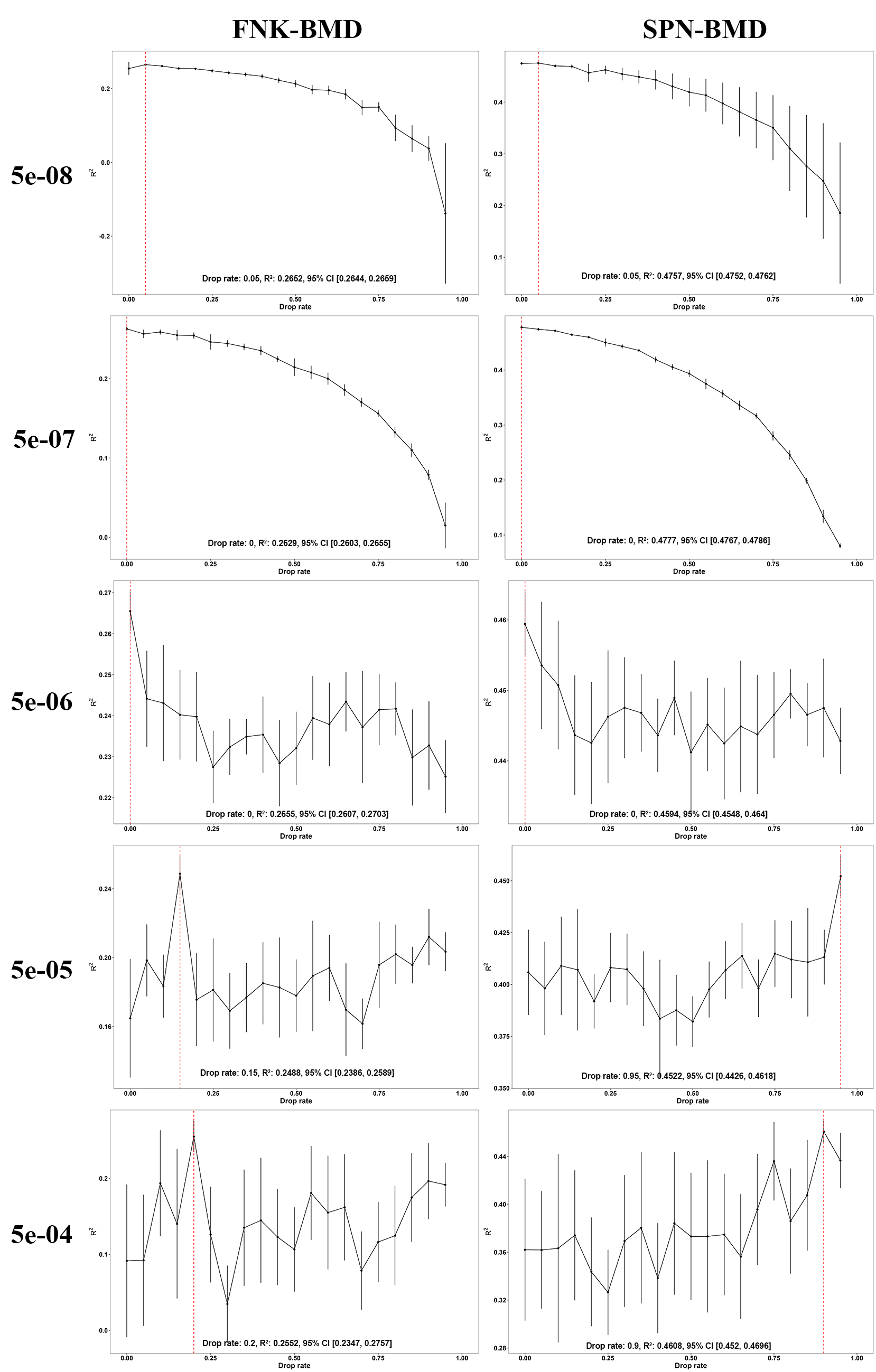

Supplement: S3 Fig — The R2 (coefficient of determination) were calculated within the UKBB Model Selection set. (TIF) [file pmed.1004451.s009.tif]

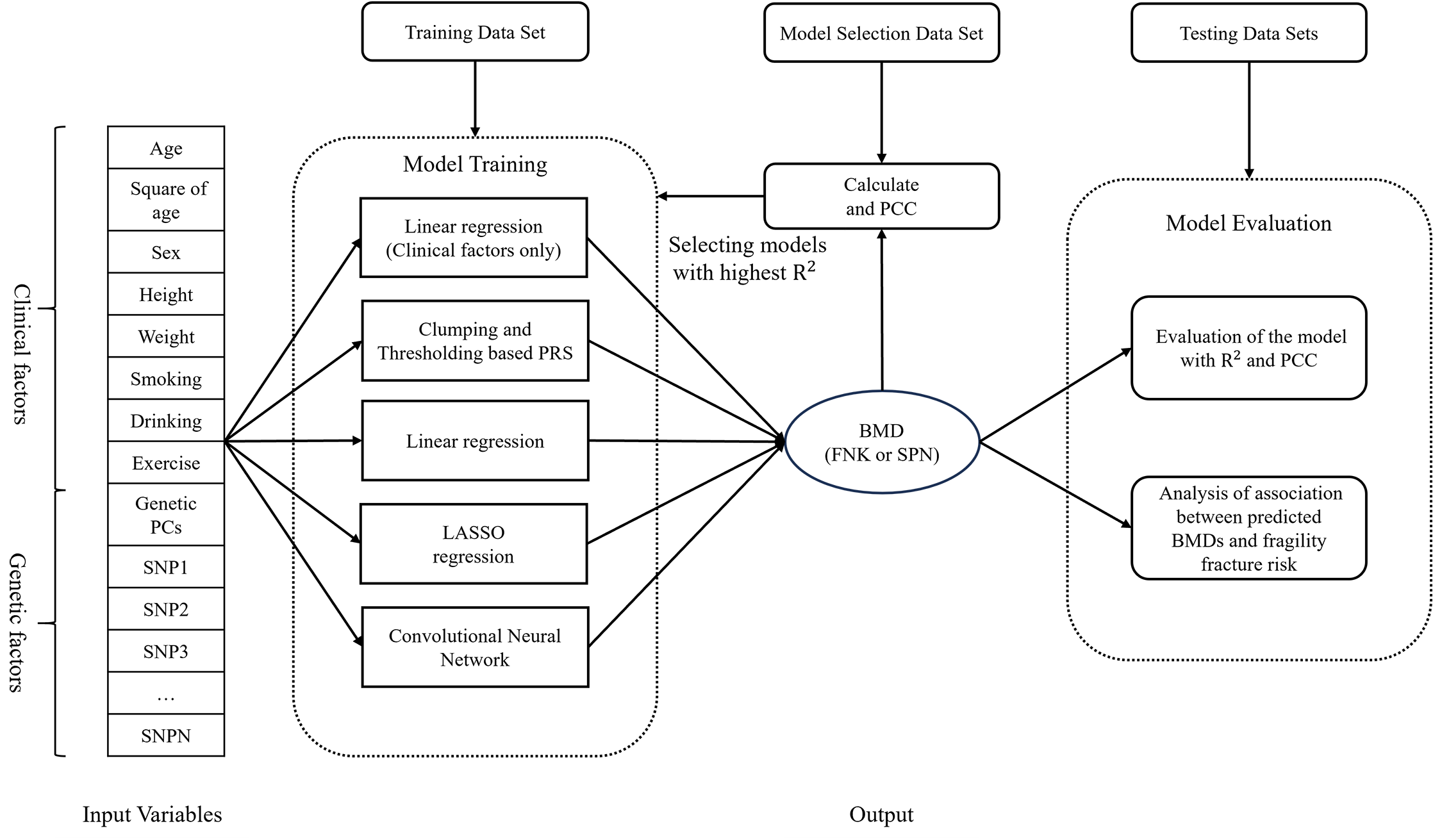

Supplement: S4 Fig — PRS, polygenic risk score; LASSO, regression with least absolute shrinkage and selection operator; R2, the coefficient of determination; PCC, Pearson correlation coefficient. (TIF) [file pmed.1004451.s010.tif]

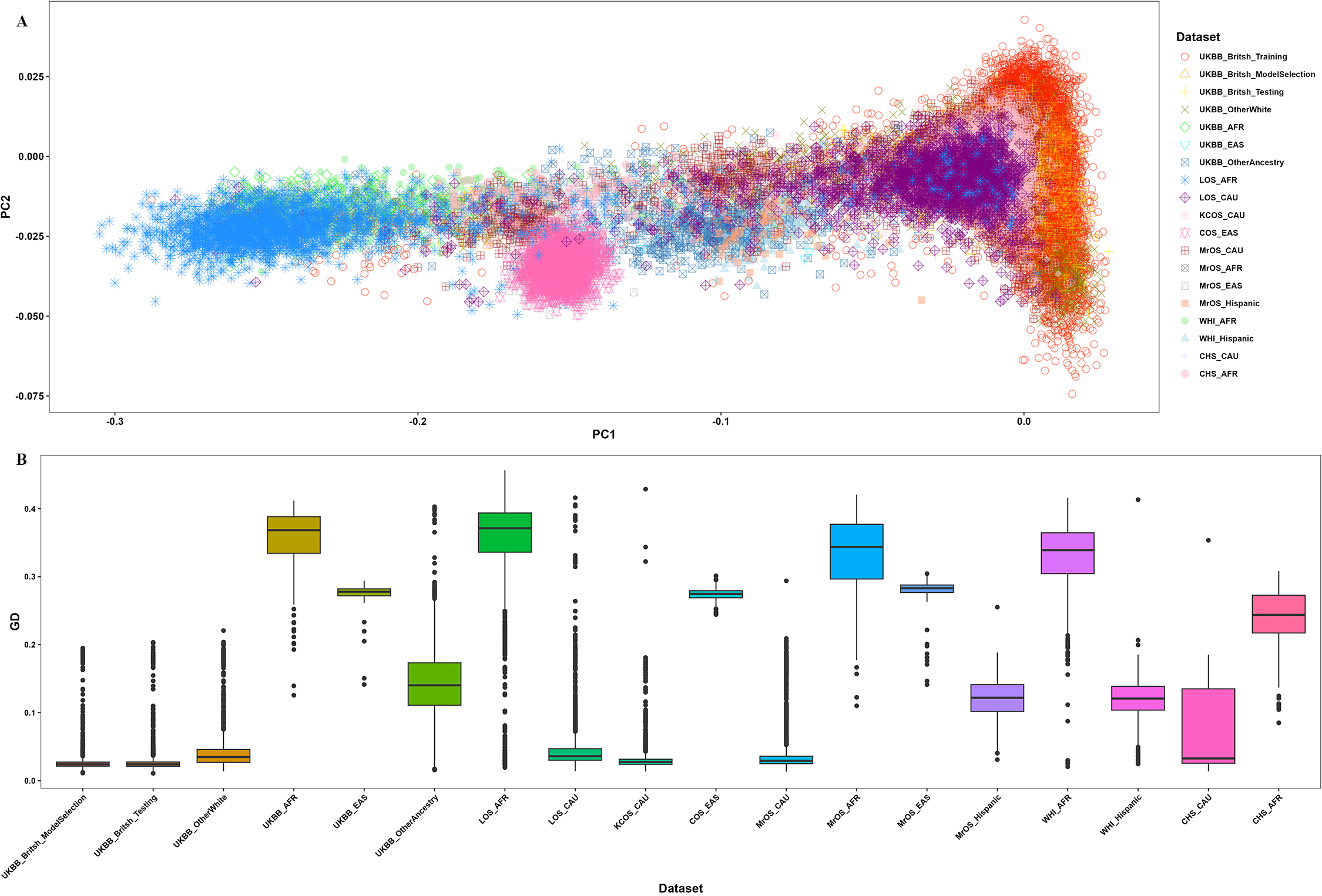

Supplement: S5 Fig — PC, principal component of genetic profile; GD, individual-level genetic distance. (TIF) [file pmed.1004451.s011.tif]
