# Supplementary material for: Variability in performance of genetic-enhanced DXA-BMD prediction models across diverse ethnic and geographic populations: A risk prediction study
Source: PLoS Med. 2024 Aug 30;21(8):e1004451. doi: 10.1371/journal.pmed.1004451 (PMC11404845; doi:10.1371/journal.pmed.1004451)
Supplement: S1 Appendix — (DOCX) [file pmed.1004451.s012.docx]

**Study cohorts and data preprocessing**

The UKBB is a large-scale biomedical database and research resource containing genetic, lifestyle and health information from more than 500,000 UK participants, enrolled at ages from 40 to 69 [1]. In this study, we extracted the participants who underwent the DXA measurement to develop the bone mineral density (BMD) prediction models. In total, 32,999 independent participants with DXA-BMD measurements in UKBB were used for model training and evaluation in our study. To validate the association between predicted BMDs and fragility fracture risk, we applied our prediction approaches and evaluated the association between the predicted BMDs and incidence of fragility fracture in 287,183 participants (17,490 fragility fracture cases and 269,693 controls) without DXA-BMD in UKBB. The UKBB participants were genome-wide genotyped using Affymetrix arrays, and their genotypes were imputed to the Haplotype Reference Consortium (HRC) reference and the UK10K panel [2, 3]. As the UKBB did not record the kinship between participants, we performed the family relationship inference in the extracted participants using the KING software [4]. KING can accurately separate unrelated pairs from close relatives, and its accuracy extends up to 3rd- or 4th-degree relationships for certain analyses, and up to 2nd-degree for kinship analysis. The tool is robust to a number of realistic scenarios, including the presence of population structure [4]. To ensure the independence of our samples, the kinship between each pair of individuals was inferred. We randomly retained only one individual from the inferred kinship relationships within 3rd-degree or closer, while the others were excluded.

The LOS study is an ongoing cross-sectional study with more than 17,000 subjects so far since 2011 for investigating genetic and nongenetic determinants of osteoporosis and other complex diseases/traits. In total, we included 2,863 Caucasians and 2,097 African Americans randomly selected (stratified by sex and race groups) from the whole LOS cohort[5]. Trained and certified research staff measured femoral neck (FNK) or lumbar spine (SPN) BMDs for each subject with DXA (Hologic QDR-4500 Discovery DXA scanner, Hologic Inc., Bedford, MA, USA) in all subjects. The isolated DNA from blood samples was sequenced at an average read depth of 22× using a BGISEQ-500 sequencer (BGI Americas Corporation, Cambridge, MA, USA) to generate two sequencing runs of paired-end 350 bp reads. For the processing of WGS data, we adhered to the GATK Best Practices for Germline SNPs & Indels workflow (https://gatk.broadinstitute.org), which facilitated the identification and calling of single-nucleotide variants (SNVs) and small insertion–deletion mutations (Indels). The workflow commenced with the HaplotypeCaller tool, which simultaneously identified SNPs and Indels, allowing for the resolution of allele balance discrepancies through local de novo haplotype assembly in targeted regions. Subsequently, we utilized the GenotypeGVCFs tool to perform joint genotyping across multiple samples and to annotate the variants using the Single Nucleotide Polymorphism Database (dbSNP) V138 resource [6]. Rather than imposing a predetermined coverage threshold for variant filtering, we adopted the Variant Quality Score Recalibration (VQSR) method. VQSR is a sophisticated filtering technique that employs machine learning to predict variant features from a training dataset, integrating annotations such as sequencing depth, frameshifts, read orientation, and position. This method affords researchers the flexibility to optimize the trade-off between sensitivity and specificity in variant detection. VQSR is highly endorsed within GATK for its capacity to enhance the accuracy and reliability of variant identification.

The KCOS study included 2,271 unrelated subjects of European ancestry from the Kansas City osteoporosis study (KCOS_CAU). The COS study included 1,569 unrelated subjects of East Asian (Chinese Han) ancestry from the China osteoporosis study (COS_EAS). The subjects in these two cohorts were genotyped with the Affymetrix 500K Array. More details about the sample processing, DNA extraction as well as variant calling can be found in previous published studies [7].

The MrOS is an international multi-center longitudinal study of elderly men. The MrOS cohort recruited 5,995 men aged ≥ 65 years at multiple assessment centers in the USA between 2000 and 2002 [8]. Among them, we included participants with both genotype data and DXA-BMD measurements. The genomic data of MrOS was genotyped with the Illumina HumanOmni1-Quad_v1-0_H array.

The WHI study is a long-term national health investigation that has focused on strategies for preventing heart disease, breast and colorectal cancer, as well as osteoporotic fractures in postmenopausal women [9]. The original WHI study enrolled 161,808 postmenopausal women between 1993 and 1998. In this study, we included 1,064 individuals with both genotype data generated using the Affymetrix array and DXA-BMD measurements.

The WHI study is a long-term national health investigation that has focused on strategies for preventing heart disease, breast and colorectal cancer, as well as osteoporotic fractures in postmenopausal women [9]. In this study, we included 1,064 individuals with both genotype data and DXA-BMD measurements. Subsequently, we segregated them by ancestry: 671 individuals of Black or African-American ancestry (WHI_AFR) and 393 individuals of Hispanic/Latino ancestry (WHI_HIS).

The CHS is a prospective investigation aimed at identifying risk factors for the development and progression of coronary heart disease and stroke in individuals aged 65 years and older [10]. The original cohort, enrolled between 1989 and 1990, comprised 5,201 participants from four U.S. communities, all of whom underwent extensive clinic examinations to evaluate markers of subclinical cardiovascular disease. For this study, we included individuals with both genotype data generated using the Illumina array and DXA-BMD measurements.

**Phenotype measurements and quality control**

The BMD measurements were performed at an imaging assessment centre for UKBB with a GE-Lunar iDXA instrument. For participants with available measurements for both the left and right sides (data fields 23299 and 23208, respectively), we calculated the FNK-BMD as the average of these two values. If only one side was measured, that single value was used for the FNK-BMD. SPN-BMD (data field 23234) was determined using the L1-L4 lumbar vertebrae. The full protocol can be accessed on the UKBB website (https://biobank.ndph.ox.ac.uk/showcase/label.cgi?id=103). The clinical risk factors were collected at the imaging visit (prior to DXA scan), including age, sex, height, weight. The lifestyle variables were collected from the touchscreen questionnaire completed at the UKBB assessment centre, including smoking, drinking and exercise. The individuals in the UKBB Fracture case-control set did not attend the DXA measurement, so their variables were obtained at the initial assessment visit. Fragility fracture cases were identified based on the 10th revision of the International Statistical Classification of Diseases and Related Health Problems (ICD10) codes of primary or secondary diagnoses and self-reported codes following the initial assessment. Individuals without any hospital inpatient data were excluded from this study. We excluded fractures of the skull, face, hands and feet, pathological fractures due to malignancy, atypical femoral fractures, periprosthetic, and healed fracture codes. The individuals with any fragility fracture code within the next 10 years were defined as the fragility fracture cases, and their follow-up time to fracture occurrence was also captured. The follow-up duration was defined as the time from the initial assessment to the first occurrence of a fragility fracture or the individual’s death, up to a maximum of 10 years. In total, we identified 17,490 fragility fractures cases using ICD10 and self-reported codes, and 269,693 individuals as controls. For the Case group, the average follow-up duration was 6.54 years with a standard deviation of 3.20 years. For the Control group, the average follow-up duration was 9.80 years with a standard deviation of 1.04 years. The individuals with any fracture record (identified with ICD10 and self-reported codes) before the initial assessment were defined with previous fracture. The rheumatoid arthritis (RA) cases were identified based on ICD10 codes of primary or secondary diagnoses, and the glucocorticoid using information was obtained from the record-level primary care linked data. Individuals lacking confirmed sex or age information were excluded. For other missing clinical factors, we imputed the values using the median of the respective cohort. A full list of ICD10 and self-reported codes used can be found in S1 Table.

**Prediction models of BMDs**

In the UKBB Training set (N = 17,964), we built four prediction models that integrated with clinical and genetic factors to predict FNK-BMD and SPN-BMD, separately. These models were:

1. Clumping and Thresholding (C+T) based PRS: Initially, we derived the C+T based PRSs from the GWAS for FNK-BMD and SPN-BMD in the UKBB British Training Set using PRSice-2 [11]. C+T based PRS is a simple and widely employed for PRS calculation[12]. It involves summing individual dosages across all included SNPs, where each individual's dosage for a given SNP is multiplied by the reported GWAS effect size. Subsequently, we integrated these calculated PRS with clinical factors to train a regression model for predicting BMDs.
2. Linear regression (LR): Differing from the C+T based PRS approach, this method does not require pre-determined SNP weights from GWAS but learns them directly from the training data. This LR model can be useful for assessing the contribution of each SNP and clinical factor to the BMD [13].
3. Regression with Least absolute shrinkage and selection operator (LASSO) [14]: In contrast to a simple linear regression model, LASSO models incorporate a regularization parameter (λ) for both variable selection and regularization. Given that simple linear regression tends to overfit when dealing with a large number of input SNPs, LASSO models enhance prediction accuracy and interpretability through variable selection and regularization [14].
4. Convolutional Neural Network (CNN) [15]: The CNN model, a deep learning architecture widely used dimensionality reduction and feature recognition in image processing tasks, was employed in our study. We designed a multi-layer deep learning model comprising two 1-dimensional convolutional layers (1x4) with pooling layers to extract genetic factor information. The network architecture diagram is shown in S1 Fig. This information was then combined with clinical factors using two fully connected layers for BMD regression. Compared to traditional regression models, neural network models with activation functions [the Rectified Linear Unit (ReLU) in this study] are advantageous for capturing nonlinear relationships between SNPs and BMDs [16].

For each type of model, we fitted five models that integrated clinical factors and SNPs with p-values smaller than a chosen set of thresholds ($5\times{10}^{-8}$, $5\times{10}^{-7}$, $5\times{10}^{-6}$, $5\times{10}^{-5}$, and $5\times{10}^{-4}$). For LASSO models, we initially set the parameter λ to 50 incremental steps ranging from 0.0001 to 0.1 (S2 Fig). The λ parameter controls the strength of the penalty applied to the model’s coefficients. As λ increases, the penalty increases, which can lead to some coefficients being shrunk towards zero. This process simplifies the model by reducing the number of features, which helps in preventing overfitting and improving model performance on new data [14]. To mitigate overfitting in our CNN models, we employed a dropout strategy, which involves temporarily disabling a specified fraction of the neurons before the fully connected layer during training. This approach helps in preventing the model from becoming overly reliant on any particular set of features, thereby enhancing its generalization capabilities [17]. For each CNN model, we evaluated 20 distinct dropout rates, increasing in increments of 0.05 from 0 to 0.95 (S3 Fig). For each type of model and p-value threshold, the model with the highest R^2^ in UKBB British Model Selection set was then taken forward for testing in the testing sets. In total, we included 18 clinical factors in the prediction models: age, square of age, sex, height, weight, smoking, drinking, exercise and the first 10^th^ genetic PCs. The clinical risk factors chosen for BMD and fracture risk were determined associated with osteoporosis and osteoporotic fracture [18]. They are also the most commonly used variables in genetic studies on osteoporosis, such as sex, age, body weight, etc. Furthermore, we constructed a LR model that includes only clinical factors to evaluate whether incorporating genetic factors could improve the prediction for BMDs. The diagram that shows the structure of models is presented in S4 Fig. All continuous variables (age, square of age, height, weight and genetic PCs) were normalized with zero-mean normalization, and categorical variables (sex, smoking, drinking, exercise and SNPs) were encoded with one-hot encoding. The S2 Table provides detailed information on the input features of the models.

**Reference**

1. Sudlow C, Gallacher J, Allen N, Beral V, Burton P, Danesh J, et al. UK biobank: an open access resource for identifying the causes of a wide range of complex diseases of middle and old age. PLoS Med. 2015;12(3):e1001779. Epub 20150331. doi: 10.1371/journal.pmed.1001779. PubMed PMID: 25826379; PubMed Central PMCID: PMCPMC4380465.

2. McCarthy S, Das S, Kretzschmar W, Delaneau O, Wood AR, Teumer A, et al. A reference panel of 64,976 haplotypes for genotype imputation. Nat Genet. 2016;48(10):1279-83. Epub 2016/08/23. doi: 10.1038/ng.3643. PubMed PMID: 27548312; PubMed Central PMCID: PMCPMC5388176.

3. Consortium UK, Walter K, Min JL, Huang J, Crooks L, Memari Y, et al. The UK10K project identifies rare variants in health and disease. Nature. 2015;526(7571):82-90. Epub 2015/09/15. doi: 10.1038/nature14962. PubMed PMID: 26367797; PubMed Central PMCID: PMCPMC4773891.

4. Manichaikul A, Mychaleckyj JC, Rich SS, Daly K, Sale M, Chen WM. Robust relationship inference in genome-wide association studies. Bioinformatics. 2010;26(22):2867-73. Epub 2010/10/12. doi: 10.1093/bioinformatics/btq559. PubMed PMID: 20926424; PubMed Central PMCID: PMCPMC3025716.

5. Greenbaum J, Su KJ, Zhang X, Liu Y, Liu A, Zhao LJ, et al. A multiethnic whole genome sequencing study to identify novel loci for bone mineral density. Hum Mol Genet. 2022;31(7):1067-81. Epub 2021/10/22. doi: 10.1093/hmg/ddab305. PubMed PMID: 34673960; PubMed Central PMCID: PMCPMC8976433.

6. Smigielski EM, Sirotkin K, Ward M, Sherry ST. dbSNP: a database of single nucleotide polymorphisms. Nucleic Acids Res. 2000;28(1):352-5. doi: 10.1093/nar/28.1.352. PubMed PMID: 10592272; PubMed Central PMCID: PMCPMC102496.

7. Zhang L, Choi HJ, Estrada K, Leo PJ, Li J, Pei YF, et al. Multistage genome-wide association meta-analyses identified two new loci for bone mineral density. Hum Mol Genet. 2014;23(7):1923-33. Epub 2013/11/20. doi: 10.1093/hmg/ddt575. PubMed PMID: 24249740; PubMed Central PMCID: PMCPMC3943521.

8. Orwoll E, Blank JB, Barrett-Connor E, Cauley J, Cummings S, Ensrud K, et al. Design and baseline characteristics of the osteoporotic fractures in men (MrOS) study--a large observational study of the determinants of fracture in older men. Contemp Clin Trials. 2005;26(5):569-85. Epub 2005/08/09. doi: 10.1016/j.cct.2005.05.006. PubMed PMID: 16084776.

9. Design of the Women's Health Initiative clinical trial and observational study. The Women's Health Initiative Study Group. Control Clin Trials. 1998;19(1):61-109. Epub 1998/03/11. doi: 10.1016/s0197-2456(97)00078-0. PubMed PMID: 9492970.

10. Fried LP, Borhani NO, Enright P, Furberg CD, Gardin JM, Kronmal RA, et al. The Cardiovascular Health Study: design and rationale. Ann Epidemiol. 1991;1(3):263-76. doi: 10.1016/1047-2797(91)90005-w. PubMed PMID: 1669507.

11. Choi SW, O'Reilly PF. PRSice-2: Polygenic Risk Score software for biobank-scale data. Gigascience. 2019;8(7). Epub 2019/07/16. doi: 10.1093/gigascience/giz082. PubMed PMID: 31307061; PubMed Central PMCID: PMCPMC6629542.

12. Clark K, Leung YY, Lee WP, Voight B, Wang LS. Polygenic Risk Scores in Alzheimer's Disease Genetics: Methodology, Applications, Inclusion, and Diversity. J Alzheimers Dis. 2022;89(1):1-12. doi: 10.3233/JAD-220025. PubMed PMID: 35848019; PubMed Central PMCID: PMCPMC9484091.

13. SEAL HL. Studies in the History of Probability and Statistics. XV The historical development of the Gauss linear model. Biometrika. 1967;54(1-2):1-24. doi: 10.1093/biomet/54.1-2.1.

14. Tibshirani R. Regression Shrinkage and Selection via The Lasso: A Retrospective. Journal of the Royal Statistical Society Series B: Statistical Methodology. 2011;73(3):273-82. doi: 10.1111/j.1467-9868.2011.00771.x.

15. Alzubaidi L, Zhang J, Humaidi AJ, Al-Dujaili A, Duan Y, Al-Shamma O, et al. Review of deep learning: concepts, CNN architectures, challenges, applications, future directions. Journal of Big Data. 2021;8(1):53. doi: 10.1186/s40537-021-00444-8.

16. Ding B, Qian H, Zhou J, editors. Activation functions and their characteristics in deep neural networks. 2018 Chinese Control And Decision Conference (CCDC); 2018 9-11 June 2018.

17. Srivastava N, Hinton G, Krizhevsky A, Sutskever I, Salakhutdinov R. Dropout: a simple way to prevent neural networks from overfitting. J Mach Learn Res. 2014;15(1):1929–58.

18. Kelsey JL. Risk factors for osteoporosis and associated fractures. Public Health Rep. 1989;104 Suppl(Suppl):14-20. Epub 1989/09/01. PubMed PMID: 2517695; PubMed Central PMCID: PMCPMC1580372.
