# Supplementary material for: Variability in performance of genetic-enhanced DXA-BMD prediction models across diverse ethnic and geographic populations: A risk prediction study
Source: PLoS Med. 2024 Aug 30;21(8):e1004451. doi: 10.1371/journal.pmed.1004451 (PMC11404845; doi:10.1371/journal.pmed.1004451)
Supplement: Attachment — Submitted filename: Response.docx [file pmed.1004451.s013.docx]

Author's Response To Reviewer Comments

Dear Editor:

Thank you very much for your kind help in offering enough time for us to improve our manuscript. We have revised the manuscript point-by-point according to all of the reviewer and editorial points. We have submitted a clean version of the paper as the main article file and a version with changes marked should as a marked-up manuscript. Page and line numbering in this response refers to the clean document. We would like to thank you again for your assistance. Hopefully, our revision will meet your and the reviewers' requirements.

To reviewer:

We are very grateful for the comments you provided about our manuscript “The performance of genetic-enhanced DXA-BMD predicting models trained in UK biobank varies across diverse ethnic and geographical populations”. Your suggestions are very valuable to us, both for composing the manuscript and for our further research. Along with reviewing the comments, our team conducted analyses and then addressed all the comments in the manuscript. The revision is being submitted with the hope that they can be approved.

Please find our response below.

**Editorial comments:**

**1) The editorial team are in agreement that your manuscript is very interesting and well presented. Overall, opinions were somewhat split because of the limited direct clinical application. This was echoed by the Academic Editor (please see below). Please consider this point as part of your revisions and ensure to include clear discussion, not only of the potential usefulness of such a model, but the benefits and barriers to its implementation in clinical practice.**

Response: Thank you for your reminder. We have responded this below. We added some contents in the Discussion section, including the usefulness of such models, the potential benefits and barriers to its implementation in clinical practice at current. The corresponding modifications are as follows (Page 25-26, Line 678-697):

*“Therefore, for areas where DXA measurement is inconvenient, obtaining genetic information may be more convenient and cost-effective. Moreover, building prediction models based on genetic information may be particularly useful in this situation, because the one-time cost of genotyping can be used not only for evaluation of osteoporosis but also for prediction of other complex diseases (56-59). For instance, such genetic-factor-based prediction approaches have proven effective for diseases such as atrial fibrillation, coronary artery disease, Type 2 diabetes, breast cancer, colorectal cancer, and prostate cancer (21, 59-63). Furthermore, it has been reported that genetic pre-screening could reduce the number of screening tests needed to identify individuals at risk of osteoporotic fractures, potentially enhancing the efficiency of osteoporosis screening programs (64). An additional benefit of disease risk prediction models based on genetic information is the ability to consider early identification of high-risk individuals in primary prevention strategies. It is imperative to highlight that the predictive models should serve as tools to support, not replace, the clinical judgment of physicians and the discernment of individuals. Decisions regarding clinical examinations or proactive interventions should not be made on the predictive outcomes alone. Vigilance against disease risks must be maintained, even if the model indicates a lower probability of illness. A realistic expectation for our approach is to identify individuals who have a higher risk of osteoporosis or fractures compared to the general populace, thereby encouraging them to recognize potential health threats, embrace positive lifestyle changes, and pursue timely diagnosis and preventive measures.”*

**2) Data Availability**

**PLOS Medicine requires that the de-identified data underlying the specific results in a published article be made available, without restrictions on access, in a public repository or as Supporting Information at the time of article publication, provided it is legal and ethical to do so.** **Please see the policy at http://journals.plos.org/plosmedicine/s/data-availability and FAQs at**

**http://journals.plos.org/plosmedicine/s/data-availability#loc-faqs-for-data-policy**

**The Data Availability Statement (DAS) requires revision. For each data source used in your study:**

**a) If the data are freely or publicly available, note this and state the location of the data: within the paper, in Supporting Information files, or in a public repository (include the DOI or accession number).**

**b) If the data are owned by a third party but freely available upon request, please note this and state the owner of the data set and contact information for data requests (web or email address). Note that a study author cannot be the contact person for the data.**

**c) If the data are not freely available, please describe briefly the ethical, legal, or contractual restriction that prevents you from sharing it. Please also include an appropriate contact (web or email address) for inquiries (again, this cannot be a study author).**

Response: Thank you for your reminder. This study incorporates datasets from various sources. Data from the UK biobank, the Osteoporotic Fractures in Men Study (MrOS), the Women's Health Initiative Clinical Trial and Observational Study (WHI) and the Cardiovascular Health Study study (CHS) are Third-party data. As we do not have the rights to share them directly, descriptions of these datasets and their respective third-party sources are detailed in the Data Availability section. The rest data, including data from the Louisiana Osteoporosis Study (LOS), the Kansas City Osteoporosis Study (KCOS), the China Osteoporosis Study (COS), are in-house data. We are going to upload the raw whole genome array or sequencing (WGS) data to AgingReserchBiobank (https://agingresearchbiobank.nia.nih.gov/). However, due to ethical and legal considerations, as well as the substantial size of the WGS data, this process may take several months. As an alternative, we uploaded a reduced dataset containing GWAS results, genotypes of clumped SNPs, and phenotypes participants included in this study, all of which have been anonymized. Currently, the raw genotype WGS and array data are available upon reasonable request from the corresponding author. Theses Data Availability statements are included in the manuscript (Page 33, Line 881-893):

*“*

*The UK Biobank data utilized in this study can be accessed through an application to the UK Biobank Resource at https://www.ukbiobank.ac.uk;*

*Data from the Osteoporotic Fractures in Men Study (MrOS, accession id: phs000373.v1.p1), the Women's Health Initiative Clinical Trial and Observational Study (WHI, accession id: phs000200.v12.p3) and the Cardiovascular Health Study study (CHS, accession id: phs000287.v7.p1) can be requested via the database of Genotypes and Phenotypes (dbGaP) at https://www.ncbi.nlm.nih.gov/gap.*

*The anonymized, lightweight dataset from the Louisiana Osteoporosis Study (LOS), the Kansas City Osteoporosis Study (KCOS), the China Osteoporosis Study (COS)) could obtained at Mendeley Data (https://data.mendeley.com/datasets,* *Doi: 10.17632/p78t84md5h.1). The raw whole genome array or sequencing (WGS) data is available from the corresponding author on reasonable request.*

*”*

**3) Of all authors who submit modelling studies we ask that the following points are included in the main manuscript. Please review the list below and ensure that each item is included:**

***** **Please provide a** **diagram** **that shows the model structure, including how the disease natural history is represented, the process and determinants of disease acquisition, and how the putative intervention could affect the system.**

Response:

Thanks for pointing that out. We’ve added a diagram that shows the model structure, including the input and output, please see the S4 Fig. The citations of this figure has been included in the Methods section of main manuscript (Page 17, Line 471):

*“****The diagram that shows the structure of models is presented in S4 Fig.****”*

***** **Please provide a complete list of model parameters, including clear and precise descriptions of [the meaning of each parameter, together with the values or ranges for each, with justification or the primary source cited, and important caveats about the use of these values noted].**

Response:

Thanks for pointing that out. We have added the descriptions of model parameters follows in the models description section in Methods (Page 17, Line 451-464):

*“****For each type of model, we fitted five models that integrated clinical factors and SNPs with p-values smaller than a chosen set of thresholds (***$5\times{10}^{-8}$**,** $5\times{10}^{-7}$**,** $5\times{10}^{-6}$**,** $5\times{10}^{-5}$**, *and*** $5\times{10}^{-4}$***). For LASSO models, we initially set the parameter λ to 50 incremental steps ranging from 0.0001 to 0.1 (S2 Fig). The λ parameter controls the strength of the penalty applied to the model’s coefficients. As λ increases, the penalty increases, which can lead to some coefficients being shrunk towards zero. This process simplifies the model by reducing the number of features, which helps in preventing overfitting and improving model performance on new data (42). To mitigate overfitting in our CNN models, we employed a dropout strategy, which involves temporarily disabling a specified fraction of the neurons before the fully connected layer during training. This approach helps in preventing the model from becoming overly reliant on any particular set of features, thereby enhancing its generalization capabilities (45). For each CNN model, we evaluated 20 distinct dropout rates, increasing in increments of 0.05 from 0 to 0.95 (S3 Fig). For each type of model and p-value threshold, the model with the highest R^2^ in UKBB British Model Selection set was then taken forward for testing in the testing sets.****”*

Added a reference:

*“****45****.	Srivastava N, Hinton G, Krizhevsky A, Sutskever I, Salakhutdinov R. Dropout: a simple way to prevent neural networks from overfitting. J Mach Learn Res. 2014;15(1):1929–58.”*

*** Please provide a clear statement about how the model was fitted to the data [including goodness-of-fit measure, the numerical algorithm used, which parameter varied, constraints imposed on parameter values, and starting conditions].**

Response:

Thanks for pointing that out. We have added a statement about how the model was fitted to the data in the ‘Model training and evaluation’ section in the Methods (Page 18, Line 478-489):

*“****The model training and evaluation was performed separately for FNK-BMD and SPN-BMD. For each type of model, we trained the model in the UKBB British Training Set using a series of parameters (See the Prediction models of BMDs section). All weights in the models were initialized using random initialization, and the model parameters were adjusted based on the Mean Squared Error (MSE) (46). Due to the large total number of variables and sample size, it is not feasible to process all data at once. Therefore, we have adopted the Mini-Batch Gradient Descent (MBGD) approach (47), with a batch size set to 32 and an initial learning rate of*** ${10}^{-4}$***, utilizing the Adam optimizer (48) to adjust the learning rates of the parameters. We optimized the models by minimizing the MSE, and evaluated the models with the coefficient of determination (R2) and Pearson correlation coefficient (PCC) between the predicted and actual BMD values. The model conduction and evaluation were performed using Python3.9 and TensorFlow2.****”*

Added References:

*“*

*46.	Mean Squared Error. The Concise Encyclopedia of Statistics. New York, NY: Springer New York; 2008. p. 337-9.*

*47.	Ruder S. An overview of gradient descent optimization algorithms. ArXiv. 2016;abs/1609.04747.*

*48.	Kingma D, Ba J. Adam: A Method for Stochastic Optimization. Computer Science. 2014.*

*”*

*** For uncertainty analyses, please state the sources of uncertainties quantified and not quantified [can include parameter, data, and model structure].**

***** **Please provide sensitivity analyses to identify which parameter values are most important in the model. Uncertainty estimates seek to derive a range of credible results on the basis of an exploration of the range of reasonable parameter values. The choice of method should be presented and justified.**

Response:

Thanks for pointing that out. For uncertainty and sensitivity analyses, we employed a bootstrap strategy, randomly resampling the training dataset 50 times to conduct separate training and testing. This approach is utilized to estimate the mean and variance of the model’s performance. The advantage of this method lies in its independence from strict parametric assumptions, making it applicable to a variety of statistical issues, such as nonlinear regression, confidence interval estimation, and bias assessment.

We have added a description about this in the Methods section (Page 18-19, Line 491-497):

*“*

***Uncertainty and sensitivity analyses***

***To estimate the bias and confidence intervals of model performances, we implemented a bootstrap strategy, randomly resampling the training dataset 50 times for independent training and testing. The bootstrap method is particularly robust, providing estimates of the model’s mean performance and variability. Its key advantage lies in its non-reliance on rigid parametric assumptions, offering flexibility in addressing a range of statistical issues, such as nonlinear regression, determination of confidence intervals, and evaluation of bias (49)..***

*”*

Added References:

*“49.	Yu H. Bootstrap. In: Liu L, Özsu MT, editors. Encyclopedia of Database Systems. New York, NY: Springer New York; 2018. p. 335-6.”*

*** Please discuss the scientific rationale for this choice of model structure and identify points where this choice could influence conclusions drawn. Please also describe the strength of the scientific basis underlying the key model assumptions.**

Response:

Thanks for pointing that out. The primary scientific rationale for our choice to combine genetic and clinical factors in predicting BMD is that BMD is influenced by both these elements. As for the selection of the final prediction model, it is data-driven; that is, we construct a series of models with varying parameters, test them in a model selection dataset independent of the training dataset, and choose the model with the best performance. We have added a discourse about the choice of model structure in the Discussion section (Page 27, Line 728-739):

*“Our model concurrently accounts for both genetic and clinical factors, which distinguishes it from existing PRS research. Current PRS research only accounts for the heritable component of a trait and ignores the significant role of environmental and lifestyle factors in disease etiology, further limiting their overall prediction power. The primary scientific rationale for our choice to integrate genetic and clinical factors in predicting BMD is that BMD is influenced by both these elements. For instance, we included clinical factors such as sex, age, height, and weight, which have been proven to be closely associated with BMD (11). Regarding genetic factors, we incorporated SNPs associated with BMD based on GWAS results. Through a data-driven process, we established the most suitable predictive models for FNK- and SPN-BMD in our study. Of course, as mentioned earlier, these model selections are contingent on specific traits and the existing training dataset.”*

**Items are derived from Geoffrey P Garnett, Simon Cousens, Timothy B Hallett, Richard Steketee, Neff Walker. Mathematical models in the evaluation of health programmes. (2011) Lancet** **DOI:10.1016/S0140-6736(10)61505-X**

**4) Statistical reporting**

**Throughout, including tables and figures, please quantify the main results with 95% CIs and p values.**

**When reporting p values please report as <0.001 and where higher as p=0.002, for example. If not reporting p values, for the purpose of transparent data reporting, please clearly state the reasons why not. When reporting 95% CIs please separate upper and lower bounds with commas instead of hyphens as the latter can be confused with reporting of negative values.**

**Please include the actual amounts and/or absolute risk(s) of relevant outcomes (including NNT or NNH where appropriate), not just relative risks or correlation coefficients. (example for absolute risks: PMID: 28399126).**

Response:

Thanks for your reminder. As previously mentioned, we employed a bootstrap strategy to estimate the bias and confidence intervals of model performances. We have quantified the main results using a 95% CI. These modifications include:

a. In the Results section, when reporting the predictive performance of the model, we have included the 95% CI for the R² :

(Page 20, Line 550-553) *“For the regression model with only clinical factors, the R^2^ of the FNK-BMD and SPN-BMD were 25.388% with a 95% confidence interval (CI) of [25.386%, 25.390%] and 45.313% (95% CI [45.312%, 45.314%]), respectively.”*

(Page 21, Line 558-565) *“For FNK-BMD, the prediction model was trained with LASSO when the threshold was set at 5×10^(-6) which resulted in an R2 of 27.70% (95% CI [27.56%, 27.84%]), indicating that the prediction model could explain 27.7% of the variance in FNK-BMD in the UKBB British Model Selection Set. Compared with the regression model with only clinical factors, the LASSO model for FNK-BMD improved by 2.3% in R2. For SPN-BMD, the prediction model was trained with LASSO when set the threshold as 5×10^(-7), which resulted in a R2 of 48.28% (95% CI [48.23%, 48.34%]), indicating that the prediction model could explain 48.28% of the variance in SPN-BMD.”*

(Page 21, Line 572-580) *“The model for predicting FNK-BMD explained 24.03% (95% CI [23.89%, 24.17%]) and 24.06% (95% CI [23.83%, 24.29%]) of variance in the UKBB British Test Set and the UKBB other White set, respectively. The model for predicting SPN-BMD explained 44.79% (95% CI [44.74%, 44.84%]) and 44.17% (95% CI [44.08%, 44.25%]) of variance, respectively. However, the model performance dropped significantly in the UKBB African ancestry set, East Asian ancestry set and other ancestry set. Especially among the UKBB African ancestry set, the R2 for FNK-BMD and SPN-BMD were -0.089 (95% CI [-0.173, -0.005]) and -0.333 (95% CI [-0.446, -0.221]), respectively, indicating that the models cannot capture any BMD variability.”*

(Page 23, Line 611-614) *“British Test Set and the UKBB other White set, respectively. The model for predicting SPN-BMD explained 44.79% (95% CI [44.74%, 44.84%]) and 44.17% (95% CI [44.08%, 44.25%]) of variance, respectively. However, the model performance dropped significantly in the UKBB African ancestry set, East Asian ancestry set and other ancestry set. Especially among the UKBB African ancestry set, the R2 for FNK-BMD and SPN-BMD were -0.089 (95% CI [-0.173, -0.005]) and -0.333 (95% CI [-0.446, -0.221]), respectively, indicating that the models cannot capture any BMD variability.”*

(Page 23, Line 625-635) *“Next, we calculated the odds ratios (OR) using the logistic regression to assess the association between the predicted BMDs and fragility fracture risk. As shown in the Table 3, the OR between predicted FNK-BMD and fragility fracture was 0.575 (95% CI [0.414, -0.797]) (P value = 0.001) in the KCOS_CAU set, indicating that for every one standard deviation increase in BMD, the risk of fracture is reduced by 42.5%. The OR between predicted SPN-BMD and fragility fracture was 0.741 (95% CI 0.589-0.930) (P value = 0.010) in the KCOS_CAU set, indicating that for every one standard deviation increase in BMD, the risk of fracture is reduced by 25.9%. The OR value between predicted SPN-BMD and fragility fracture was 0.839 (95% CI [0.736, -0.957]) (P value = 0.009) in the MrOS_CAU set, indicating that for every one standard deviation increase in BMD, the risk of fracture was reduced by 16.1%.”*

b. In Fig 2 and 3, we have added error bars to represent the 95% CI. Please see the Fig 2 and Fig 3.
c. The 95% CI of main results were also presented in Tables. Please see the Table 2 and Table 3.

**5) Abstract layout**

**Please ensure you structure your abstract using the PLOS Medicine headings (Background, Methods and Findings, Conclusions). Please combine the Methods and Findings sections into one section, “Methods and findings”.**

Response:

Thanks for your reminder. We have structured the abstract using the PLOS Medicine headings (Background, Methods and Findings, Conclusions), Please see the Abstract section (Page 2-3, Line 25-60):

*“*

***Background***

*Osteoporosis presents a significant global health challenge, compromising bone quality and elevating fracture susceptibility. While dual-energy x-ray absorptiometry (DXA) stands as the gold standard for bone mineral density (BMD) assessment and osteoporosis diagnosis, its costliness and complexity impede widespread screening adoption. Predictive modeling of BMD, leveraging genetic and clinical data, emerges as a more viable and cost-effective approach for osteoporosis and fracture risk evaluation.*

***Methods and Findings***

*We* *developed BMD prediction models for the femoral neck (FNK) and lumbar spine (SPN) using various methods within a UK Biobank (UKBB) training set comprising 17,964 individuals from the British white population. Models based on Regression with Least Absolute Shrinkage and Selection Operator (LASSO), selected based on the coefficient of determination (**R^2^) from a model selection subset of 5,973 individuals from the British white population, underwent testing on five UKBB test sets and 12 independent cohorts of diverse ancestries, totaling over 15,000 individuals. Furthermore, we assessed the correlation of predicted BMDs with fragility fractures in a distinct case-control set of over 287,000 participants lacking DXA-BMDs in the UKBB of the European white population.*

*Incorporating genetic factors marginally improved predictions, capturing an additional 2.3% variation for FNK-BMD and 3% for SPN-BMD over clinical factors alone.* *Predicted BMDs exhibited significant associations with fragility fracture risk in the European white population. Nonetheless, the predictive model's performance varied between the UKBB population of other ethnic groups and the independent cohorts.*

***Conclusions***

*Our study yields novel insights into predicting osteoporosis and fracture risk. Genetic factors enhance BMD predictive performance beyond clinical factors alone. Adjusting inclusion thresholds for genetic variants (e.g.,* $5\times{10}^{-6}$*or* $5\times{10}^{-7}$*) rather than solely considering genome-wide association study (GWAS)-significant variants may further refine the model's explanatory power for BMD variations.* *This study also underscores the imperative for training models on diverse population to bolster predictive performance across various ethnic and geographical populations.*

*”*

**6) Author summary**

**At this stage, we ask that you include a short, non-technical Author Summary of your research to make findings accessible to a wide audience that includes both scientists and non-scientists. The authors summary should consist of 2-3 succinct bullet points under each of the following headings:**

**• Why Was This Study Done? Authors should reflect on what was known about the topic before the research was published and why the research was needed.**

**• What Did the Researchers Do and Find? Authors should briefly describe the study design that was used and the study’s major findings. Do include the headline numbers from the study, such as the sample size and key findings.**

**• What Do These Findings Mean? Authors should reflect on the new knowledge generated by the research and the implications for practice, research, policy, or public health. Authors should also consider how the interpretation of the study’s findings may be affected by the study limitations. In the final bullet point of ‘What Do These Findings Mean?’, please describe the main limitations of the study in non-technical language.**

**The Author Summary should immediately follow the Abstract in your revised manuscript. This text is subject to editorial change and should be distinct from the scientific abstract. Please see our author guidelines for more information: https://journals.plos.org/plosmedicine/s/revising-your-manuscript#loc-author-summary**

Response:

Thanks for your reminder. We have added a section of ‘Author summary’ follow the Abstract in the revised manuscript (Page 4-5, Line 91-134). Please also see the following:

“***Author summary***

***Why was this study done?***

*Osteoporosis is a global public health issue, but its most commonly used diagnostic method, bone mineral density (BMD) measurement by dual-energy x-ray absorptiometry (DXA), is not suitable for large-scale population screening, especially in areas with insufficient medical resources. With the development of genome sequencing technology, using genomic data to predict disease risk seems to be a more viable and cost-effective.*

*Although some studies have applied genomic prediction methods such as polygenic risk scores to the prediction of BMD, these studies often only consider a small number of variants that reach genome-wide significance, while a large number of sub-significant variants can also affect BMD. How to set the threshold for including variants requires systematic exploration. Additionally, polygenic risk scores usually only consider genetic factors and overlook clinical factors. However, for complex diseases like osteoporosis, which are greatly influenced by environmental factors, it is very necessary to consider both genetic and clinical factors.*

*Due to the current conditions of sample collection and storage, most existing genomic prediction methods are based on populations of European ancestry, and the evaluation of their prediction effect in other ethnic populations is still limited.* *This issue has begun to be taken seriously in recent years.*

***What did the researchers do and find?***

*Using a cohort of British White ancestry in UK Biobank (UKBB), we developed BMD prediction models, which integrated the clinical and genetic factors, for the femoral neck (FNK) and lumbar spine (SPN), respectively. Both the predicted FNK- and SPN-BMDs were strongly correlated with the true BMDs (R^2^≈25% for FNK-BMD and R^2^≈45% for SPN-BMD) in British and other White ancestry in UKBB. Moreover, the predicted BMDs exhibited significant associations with fragility fracture risk in the European ancestry populations in UKBB.*

*By building and testing a series of prediction models, we determined that the most suitable P-value thresholds for FNK-BMD and SPN-BMD is different (*$5\times{10}^{-6}$ *for FNK-BMD and* $5\times{10}^{-7}$ *for SPN-BMD, respectively). However, the optimal threshold is not universally constant; rather, it depends on the specific trait and the sample size of the training data.*

*Our BMD prediction models performed well in the European population in UKBB, but their performance significantly declined in the other ancestry population in UKBB and other independent cohorts.*

***What do these findings mean?***

*We show that genetic factors could improve the performance of DXA-BMD prediction beyond that of clinical factors alone.*

*Within the same population, the clinical utility of BMD prediction models can help prioritize individuals at high risk of fragility fracture, which could allow physicians to select tailored treatments for each individual in early stages to greatly increase the chances of survival.*

*However, such genetic prediction methods should not be directly applied to other populations before their effectiveness has been rigorously evaluated, even among the same ethnic groups with different geographical backgrounds. Our findings emphasize the importance of prediction model training in diverse populations.*

”

**7) Introduction layout**

**Please address past research and explain the need for and potential importance of your study. Indicate whether your study is novel and how you determined that. If there has been a systematic review of the evidence related to your study (or you have conducted one), please refer to and reference that review and indicate whether it supports the need for your study.**

Response:

Thank you for your insightful suggestions. The related past researches have been addressed in the Introduction section, we also did some modification to clarify the potential importance and novelty of our study (Page 7-8, Line 190-208):

*“Despite these advancements, the existing PRS models have some limitations. First, they can only reflect a small part of the DXA-BMD variation because the current cohorts lack sufficient sample size. Second, some of them are based on eBMD, which is a surrogate measure of BMD derived from quantitative ultrasound (QUS) of the heel. Although eBMD is moderately correlated with DXA-BMD (18), it cannot reflect the BMD status of specific sites, which is crucial for assessing site-specific fracture risks (19). Third, non-genetic risk factors are often ignored in the current PRS studies, but, for most common diseases such as osteoporosis, unglamorous but well-established risk factors like obesity, nutrition and lifestyle may matter more than a person’s genetic background (20). Last, most of the BMD prediction models were established in European populations, and their predictive performance in other populations is still lacking evaluation (20, 21). Our study aims to address these gaps by developing prediction models that integrate genetic and clinical factors, for DXA-BMD at two clinically relevant sites: the femoral neck (FNK) and lumbar spine (SPN). These sites are not only pivotal for osteoporosis diagnosis but also serve as the most significant predictors of fracture risk (22). By leveraging a larger cohort and incorporating a broader array of genetic variants, our model seeks to provide a more accurate and comprehensive reflection of BMD variation, thereby enhancing the predictive precision for osteoporosis and associated fracture risks. Further, we tested them in multiple cohorts of diverse ancestry and geographic backgrounds to assess their application across populations.”*

Please also see the full Introduction section in the revised manuscript (Page 6-8 ,Line 150-208). Meanwhile, we add two reviews supporting some of these arguments as references:

“

*20.	Sud A, Horton RH, Hingorani AD, Tzoulaki I, Turnbull C, Houlston RS, et al. Realistic expectations are key to realising the benefits of polygenic scores. BMJ. 2023;380:e073149.*

*21.	Schwarzerova J, Hurta M, Barton V, Lexa M, Walther D, Provaznik V, et al. A perspective on genetic and polygenic risk scores-advances and limitations and overview of associated tools. Brief Bioinform. 2024;25(3).*

*”*

**8) Discussion layout**

**Please present and organize the Discussion as follows: a short, clear summary of the article's findings; what the study adds to existing research and where and why the results may differ from previous research; strengths and limitations of the study; implications and next steps for research, clinical practice, and/or public policy; one-paragraph conclusion.**

Response:

Thank you for your insightful suggestions. We have adjusted the Discussion sections, and increased the following content to explain why the results may differ from previous research (Page 27, Line 728-742):

*“Our model concurrently accounts for both genetic and clinical factors, which distinguishes it from existing PRS research. Current PRS research only accounts for the heritable component of a trait and ignores the significant role of environmental and lifestyle factors in disease etiology, further limiting their overall prediction power. The primary scientific rationale for our choice to integrate genetic and clinical factors in predicting BMD is that BMD is influenced by both these elements. For instance, we included clinical factors such as sex, age, height, and weight, which have been proven to be closely associated with BMD (11). Regarding genetic factors, we incorporated SNPs associated with BMD based on GWAS results. Through a data-driven process, we established the most suitable predictive models for FNK- and SPN-BMD in our study. Of course, as mentioned earlier, these model selections are contingent on specific traits and the existing training dataset. By highlighting the significance of clinical factors, our research provides a more comprehensive understanding of BMD prediction and the assessment of osteoporosis risk, marking a moderate advancement over existing PRS studies that consider genetic factors alone.”*

**-----------------------------------------------------------**

**Comments from the reviewers:**

**Reviewer #1: The authors present a very interesting manuscript on the use of predictive modelling combining not just genetic data, but also clinical data. This exercise is highly warranted and presents a more viable and cost-effective approach to evaluating the risk of osteoporosis and fracture susceptibility.**

**A few comments are included below:**

**- How were clinical risk factors chosen for BMD and fracture risk?**

Response:

Thank you for your valuable consideration. The clinical risk factors chosen for BMD and fracture risk were determined associated with osteoporosis and osteoporotic fracture. They are also the most commonly used variables in genetic studies on osteoporosis, such as sex, age, body weight, etc. Additionally, we included rheumatoid arthritis status and glucocorticoid use because they have been proven to be related to bone density and are included in the widely used fracture risk prediction tool Fracture Risk Assessment Tool (FRAX). We have added some content to explain how we choose these clinical risk factors(Page 17, Line 466-469):

*“The clinical risk factors chosen for BMD and fracture risk were determined associated with osteoporosis and osteoporotic fracture (10). They are also the most commonly used variables in genetic studies on osteoporosis, such as sex, age, body weight, etc.”*

**-** **Was LS BMD available for L1-L4 or L2-L4?**

Response:

Thank you for your valuable consideration. The LS (Spine) BMD was assessed across the L1-L4 lumbar vertebrae (Please the the report of UK biobank: <https://biobank.ndph.ox.ac.uk/showcase/field.cgi?id=23234>), as detailed in the UK Biobank report (Field ID: 23234). We have clearly addressed this in our manuscript (Page 13, Line 349-354):

*“For participants with available measurements for both the left and right sides (**data fields 23299 and 23208, respectively), we calculated the the FNK-BMD as the average of these two values.* *If only one side was measured, that single value was used for the FNK-BMD. SPN-BMD (data field 23234) was determined using the L1-L4 lumbar vertebrae. The full protocol can be accessed on the UKBB website (“https://biobank.ndph.ox.ac.uk/showcase/label.cgi?id=103”)”*

**- Could the low predictive modelling be due to the utilisation of GWAS data and inclusion of common variants?**

Response:

Thank you for your insightful review and suggestions. We wholeheartedly agree with your viewpoint that the reduced predictive performance of the BMD prediction models in diverse populations may be linked to the GWAS data and SNPs included. The utilization of GWAS serves as a foundational step in identifying variants linked to BMD. Given the extensive number of variants present within the genome, it is not feasible to incorporate every single one into our model. Nonetheless, we recognize that GWAS may introduce biases, especially since it is intrinsically connected to the genetic background of the population. In our study, we performed GWAS using the UKBB training dataset and selected SNPs for inclusion in the models based on this. Indeed, we took into account not only common variants (Minor Allele Frequency, MAF > 0.05) but also rare variants (MAF < 0.01). Naturally, reduce the inclusion of low-quality genetic data, we excluded variants with a very low MAF (< 0.001). The significant genetic background differences between the UKBB training dataset and other populations, which can be seen in our genetic distance (GD) results, may affect the model’s predictive ability. We have expanded our Discussion to include this point (Page 29, Line 775-787):

*“Given the extensive number of variants present within the genome, it is impractical to incorporate all of them into the prediction models. Consequently, we initially used GWAS data to filter out variants associated with BMD. However, the considerable genetic background differences between the training dataset and other populations could be one of the reasons for the decreased predictive performance. We found that the average GD of datasets from European populations to the training dataset is significantly smaller than that from other ethnic groups. It was reported that GD is an important contributor to decay of predictive performance, which corresponds to decreased similarity in genetic profile (assessed by relatedness, linkage disequilibrium, and/or minor allele frequency differences, fixation index (Fst) and so on) between testing individuals and training data (36). However, GD cannot fully explain the reduced predictive performance, as a decrease in the predictive efficacy was also observed in datasets from other European populations with different geographical backgrounds.”*

**- Was there any particular clinical risk factor(s) included in the analysis that was driving the association? Perhaps even SNP(s) or Indels? In the case of the latter, in which genes did these fall?**

Response:

Thank you for bringing this to our attention. We have investigated the factors included in the final prediction model and their respective weights. We found that some rare variants such as rs554533790 plays a important role in the prediction models. However, this SNP was not detected in the independent datasets due to its low **minor allele frequency. And the absence of this SNP in other independent datasets will inevitably reduce the model’s predictive performance**. Furthermore, in our final FNK-BMD and SPN-BMD prediction models, both age and weight emerged as significant factors. Subsequently, we conducted a simple experiment where we established a linear regression model incorporating only clinical factors to fit the BMD linear model across various datasets. When employing a linear regression model that included only clinical factors to fit BMD, we observed notable differences in the coefficients of each clinical factor (including age and weight) across non-UKBB European populations and independent test datasets compared to the training set. These discrepancies could be another reason for the reduced predictive performance of our model in other populations. We have included this point in our Discussion section (Page 29-30, Line 787-808):

*“Additionally, the absence of certain variants can also affect the accuracy of predictive models. Such rare variants are particularly noticeable because their frequency in the population is too low to be detected. For instance, the SNP rs554533790, located in an intergenic region, appears in the 1000 Genomes dataset with a minor allele frequency of less than 0.005. In our study, rs554533790 has a minor allele frequency of approximately 0.0004 in the UKBB CAU populations, and zero in the East EAS and AFR populations. However, this variant was not detected in other independent datasets. Although rare, rs554533790 has a high weight in our FNK-BMD predictive model, even higher than age, highlighting its significance. Therefore, the absence of this SNP in other independent datasets will inevitably reduce the model’s predictive performance. Some clinical factors also contribute to performance degradation, such as age and weight, which were identified as crucial elements in our FNK- and SPN-BMD prediction models. To explore this further, we designed a straightforward experiment by developing a LR model that incorporates only clinical factors to predict BMD for each dataset. We found that that the coefficients for each clinical factor, including age and weight, exhibited considerable variability among across non-UKBB European cohorts and independent testing datasets in contrast to the training set. The most striking explanation for these variations is the disparate age distributions and sex ratios present in the datasets. Moreover, differences in diet, physical activity levels, sun exposure, and other lifestyle and environmental factors across populations can result in inconsistent impacts of these clinical factors on BMD, even among individuals from the similar ethnic background. These variances may represent another contributing factor to the diminished predictive capability of our model in different populations.”*

**- Is there a reason why predictive modelling in the UKBB Training sets seemed to have worked better for spine compared to FN BMD?**

Response:

Thank you for your insightful review. Predictive modeling seemed to have worked better for spine (SPN) compared to femoral neck (FNK) BMD may due to several reasons: First, The SPN and FNK have different biological and structural characteristics that might influence the accuracy of BMD predictions. The SPN is composed of multiple vertebras, which may provide a more composite and potentially more stable measure of BMD than the single measurement at the FNK. Second, the relative contributions of genetic and clinical factors vary across skeletal sites. For instance, FNK-BMD is more sensitive to the clinical effects such as physical activity and nutritional intake, while SPN-BMD is more sensitive to body weight. Although our model incorporated exercise as a factor of physical activity, it did not include nutritional intake. Furthermore, since physical activity and nutritional intake are more challenging to quantify compared to measurable values such as height and weight, the contribution of the exercise we included to the prediction of BMD is relatively low.

We have expanded our Discussion to include this point (Page 28, Line 752-767):

*“We observed an obvious difference in the explanation in variance of FNK-BMD and SPN-BMD by the prediction model. The prediction model could explain ~28% of the variance in FNK-BMD while ~48% of the variance in SPN-BMD. This may be due to several reasons: First, The SPN and FNK have different biological and structural characteristics that might influence the accuracy of BMD predictions. The SPN is composed of multiple vertebras, which may provide a more composite and potentially more stable measure of BMD than the single measurement at the FNK (65). Second, the relative contributions of genetic and clinical factors vary across skeletal sites (66). For instance, FNK-BMD is more sensitive to the clinical effects such as physical activity and nutritional intake, while SPN-BMD is more sensitive to body weight (11, 67-69).”*

Meanwhile, we added a reference:

“*67.	Piroska M, Tarnoki DL, Szabo H, Jokkel Z, Meszaros S, Horvath C, et al. Strong Genetic Effects on Bone Mineral Density in Multiple Locations with Two Different Techniques: Results from a Cross-Sectional Twin Study. Medicina (Kaunas). 2021;57(3).*

*68.	Videman T, Levalahti E, Battie MC, Simonen R, Vanninen E, Kaprio J. Heritability of BMD of femoral neck and lumbar spine: a multivariate twin study of Finnish men. J Bone Miner Res. 2007;22(9):1455-62.*

*69.	Vuillemin A, Guillemin F, Jouanny P, Denis G, Jeandel C. Differential influence of physical activity on lumbar spine and femoral neck bone mineral density in the elderly population. J Gerontol A Biol Sci Med Sci. 2001;56(6):B248-53.”*

**- In the case of WGS, were variants annotated? If yes, which tool was used? Was allele balance accounted for? What was the** **coverage cut-off used for the variants?**

Response:

Thank you for your valuable consideration. For the processing of whole-genome sequencing (WGS) data, we followed the GATK Best Practices for Germline SNPs & Indels workflow to call variants. In this process, variants were annotated with the dbSNP V138 resource using the GenotypeGVCFs tool. The allele balance issues were processed with the HaplotypeCaller tool, which is capable of calling SNPs and indels simultaneously via local de-novo assembly of haplotypes in an active region. We did not set a fixed coverage cut-off for variant filtering. Instead, we employed the Variant Quality Score Recalibration (VQSR) filtering method. VQSR is an advanced filtering technique that leverages machine learning to model variant features in a training set, incorporating variant annotations such as sequencing coverage, frameshifts, read direction, and read position. This approach enables researchers to fine-tune the balance between sensitivity and specificity in variant filtering. VQSR is highly recommended in GATK for its ability to improve the precision and dependability of variant calling.

We have refined the description in the Methods section to enhance clarity (Page 10-11 , Line 281-295):

*“For the processing of WGS data, we adhered to the GATK Best Practices for Germline SNPs & Indels workflow (https://gatk.broadinstitute.org), which facilitated the identification and calling of single-nucleotide variants (SNVs) and small insertion–deletion mutations (Indels). The workflow commenced with the HaplotypeCaller tool, which simultaneously identified SNPs and Indels, allowing for the resolution of allele balance discrepancies through local de novo haplotype assembly in targeted regions. Subsequently, we utilized the GenotypeGVCFs tool to perform joint genotyping across multiple samples and to annotate the variants using the Single Nucleotide Polymorphism Database (dbSNP) V138 resource (32). Rather than imposing a predetermined coverage threshold for variant filtering, we adopted the Variant Quality Score Recalibration (VQSR) method. VQSR is a sophisticated filtering technique that employs machine learning to predict variant features from a training dataset, integrating annotations such as sequencing depth, frameshifts, read orientation, and position. This method affords researchers the flexibility to optimize the trade-off between sensitivity and specificity in variant detection. VQSR is highly endorsed within GATK for its capacity to enhance the accuracy and reliability of variant identification.”*

Meanwhile, we added a reference:

“*32.	Smigielski EM, Sirotkin K, Ward M, Sherry ST. dbSNP: a database of single nucleotide polymorphisms. Nucleic Acids Res. 2000;28(1):352-5.”*

Thank you for all the insightful review and suggestions. We hope that our revisions have addressed your concerns and improved the quality of our manuscript.

**Reviewer #2: "The performance of genetic-enhanced DXA-BMD predicting models trained in UK biobank varies across diverse ethnic and geographical populations" reports the outcome of developing various prediction models, towards early detection of osteoporosis in the neck and spine. The focus on genetic data is in response to DXA-based bone mineral density measurement (BMD) being relatively inaccessible, and possibly inappropriate for general screening due to proportion of negative cases. The models were developed on largely white individuals between 40-69 years from the UKBB dataset, and validated on a number of independent cohorts of varying ethnicity distributions. It was found that while prediction was improved by the inclusion of genetic data, performance on non-white ethnicity cohorts was significantly poorer than on the white demographic as used to develop the prediction models.**

**This paper presents justified support for the integration of suitable genetic data in prediction models for osteoporosis screening, and raises timely concerns about such models possibly not being directly applicable to demographics outside that which were used in their development. A number of issues might be considered:**

**1. In Line 154, it is stated that "Then, we selected the model with best performance and assessed the predictive performance in three UKBB test sets and 12 independent cohorts". It might be clarified whether this "model with best performance" is separately selected for the FNK and SPN classes, or otherwise.**

Response:

Thank you for your valuable consideration. The models with best performance were separately selected for the FNK and SPN. To clarify this point, we have added a sentence to clarify this in the Results section (Page 18, Line 478):

*“The model training and evaluation was performed separately for FNK-BMD and SPN-BMD.”*

**2. Related to the above, the best model (from the four) might be indicated for the main results in Table 2. Moreover, the full results for all four models on both tasks might be reported if possible.**

Response:

Thank you for your suggestions. Certainly, we have added two supplementary tables (S3 Table and S4 Table) to document the predictive efficacy of all four models across every test dataset. Additionally, in the Results section, we simply summarized the results of this aspect (Page 22, Line 582-586):

*“Additionally, for each type of models, we selected the one with the highest R2 from the UKBB Model selection set for testing across all test datasets. The detailed outcomes are presented in S3 Table and S4 Table. Similar to the LASSO-based models, these models demonstrated better performance in the in the UKBB British Test Set and the UKBB other White set, while the performance varied significantly across other populations.*”

**3. In Line 171, it is stated that family relationship inference was performed using** **KING software. The reliability of such inference might be briefly stated.**

Response:

Thank you for your valuable advice. We have added a brief statement for reliability of the family relationship inference using KING software in the Methods section (Page 10 , Line 256-258):

*“KING can accurately separate unrelated pairs from close relatives, and its accuracy extends up to 3rd- or 4th-degree relationships for certain analyses, and up to 2nd-degree for kinship analysis. The tool is robust to a number of realistic scenarios, including the presence of population structure (31).”*

**4. In Line 172, it is stated that only individuals with no relative 3rd degree or closer were retained, to ensure sample independence. It might be clarified as to what was done when pairs (or groups) of individuals with close relatives were found. Were all of them excluded, or were one of such pairs (or groups) retained?**

Response:

Thank you for your valuable consideration. For relative individuals within 3rd degree or closer, we retained one and excluded the others. To clarify this point, we have optimized the description accordingly in the Methods section (Page 10, Line 259-260):

*“To ensure the independence of our samples, we retained only one individual from the inferred kinship relationships within 3rd-degree or closer, while the others were excluded.”*

**5. In the Phenotype measurements and quality control section, the treatment of missing variables (if any; i.e. imputation, exclusion, etc.) might be briefly stated, if relevant.**

Response:

Thank you for your valuable advice. In our study, individuals lacking confirmed sex or age information were excluded. For other missing clinical factors, we imputed the values using the median of the respective cohort. We have stated this in the Methods section (Page 14, Line 375-377):

*“Individuals lacking confirmed sex or age information were excluded. For other missing clinical factors, we imputed the values using the median of the respective cohort****.****”*

**6. In Line 245, the** **1/1000 percentile is referred to. It might be clarified as to how this threshold was chosen. Also, it might be confirmed as to whether this is 1/1000 (i.e.** **0.1th percentile), or indeed the 0.001th percentile.**

Response:

Thank you for your reminder. The the 1/1000 percentile is referred to the 0.1th percentile. Considering the requirement for a substantial sample size for model training and applicability, we adopted a stringent outlier exclusion criterion. Specifically, we excluded samples with BMD values below or above the 0.1th percentile, thus eliminating potential erroneous values that could be attributed to manual handling factors. We have optimized the description accordingly in the Methods section (Page 10, Line 261-265):

*“****Considering the requirement for a substantial number of samples for the model training and promotion, we have adopted a relatively stringent criterion for the exclusion of outlier samples. Specifically, we exclude samples with extreme BMD values below or above the 0.1th percentile to mitigate potential errors that may arise from handing factors.****”*

**7. In Line 296, it is stated that the most significant SNP was chosen within each LD window. Was there any reason to restrict each LD window to a single SNP?**

Response:

Thank you for your valuable consideration. The primary reason for this approach is the issue of sample size. Although we have utilized the largest available single ancestry DXA-BMD population to train our model, it still only encompasses a magnitude of tens of thousands, which is relatively small compared to the vast number of genomic variations. Additionally, there is a high correlation between linked SNPs. Including all of them in the model could lead to multicollinearity issues, potentially making the model difficult to train and generalize. We have added some content in the Methods to explain this point (Page 15, Line 413-414):

*“****Although we have utilized the largest available single ancestry DXA-BMD population to train our model, it still only encompasses a magnitude of tens of thousands, which is relatively small compared to the vast number of genomic variations. Additionally, there is a high correlation between linked SNPs. Including all of them in the model could lead to multicollinearity issues, potentially making the model difficult to train and generalize.****”*

**8. In Line 317, a CNN model is described (also in S1 Fig). It might be clarified as to the input dimensions of the model - is it 1D or 2D, and if the latter, what would be the width & height dimensions of the input?**

Response:

Thank you for your valuable advice. The CNN model employs a one-dimensional (1D) convolution layers (1x4). We have redrawn the model’s architectural diagram and updated the legend to present the structure of the model more clearly. We also updated the descriptions about the CNN model structure in Methods section (Page 16, Line 444-446):

*“****We designed a multi-layer deep learning model comprising two 1-dimensional convolutional layers (****1x4****) with pooling layers to extract genetic factor information. The network architecture diagram is shown in S1 Fig.****”*

**9. A number of minor grammatical/phrasing issues might be considered, e.g.**

(Page 10, Line 254) "in other ethnic population" -> "populations"

(Page 10, Line 259) "The rest participants" -> "The rest of the participants"

(Page 10, Line 418) "To reduce of the input features" -> "To reduce the input features"

etc.

Response:

Thank you for your thorough review. We have made the corresponding modifications:

(Page 12, Line 334) "in other ethnic population" -> " in other ethnic populations":

*“Due to the current conditions of sample collection and storage, most existing genomic prediction methods are based on populations of European ancestry, and the evaluation of their prediction effect in other ethnic populations is still limited.”*

(Page 12, Line 338) "The rest participants" -> "The rest of the participants":

*“The rest of the participants with DXA-BMD measurements in UKBB were divided to UKBB other White set (N = 1,960), UKBB African ancestry set (N = 218), UKBB East Asian ancestry set (N = 112) and UKBB other ancestry set (N = 795).”*

(Page 15, Line 418) "To reduce of the input features" -> "To reduce the input features"

*“To reduce the input features of prediction models, we obtained LD independent associations using PLINK1.9 by clumping SNPs in LD at a squared correlation (r^2^) > 0.05 and selected the most significant SNP from within each LD window.”*

Thank you for all the valuable reminders and suggestions. We hope that our explanation has addressed your concerns and improved the quality of our manuscript.

**Reviewer #3: In this study, the authors developed a predictive model for dual-energy X-ray absorptiometry (DXA)bone mineral density (BMD) at femoral neck (FNK) and lumbar spine (SPN), based on genetic and clinical factors within a Caucasian cohort from the UK Biobank (UKBB). The model was then tested across other ancestry groups within the UKBB and several independent cohorts. The findings revealed that the predictive model performed well within the UKBB Caucasian population, but there was a notable variance in performance across other populations and independent cohorts. The study demonstrated that genetic factors could improve the performance of DXA-BMD prediction beyond that of clinical factors alone. Moreover, the predicted BMDs significantly improved the prediction of fracture risk. This study provides new insights into predicting osteoporosis and fracture risk, aiding clinicians in better understanding and preventing fragile fractures, thereby improving patient care and quality of life. Additionally, testing the BMD predictive model's performance across different ethnic backgrounds underscores the importance of training models on diverse population datasets, which is scientifically substantiated for public health measures targeting prevention. I have several comments for authors to consider improve the manuscript:**

**1. To underscore the potential clinical and research implications of this study, it would be beneficial to expand upon the significance of the findings in the Conclusion section. This could include discussing how their findings might influence clinical practices or directions for future studies.**

Response:

Thank you for your insightful review and suggestions. We have expanded the Conclusion section to elucidate the implications of our findings for future research and clinical applications (Page 31, Line 851-857):

*“Our research highlights the importance of incorporating clinical factors into genomic disease risk prediction in future studies. From a clinical perspective, the value of these genomic prediction methods lies in their ability to identify high-risk individuals early. This early identification encourages individuals to take their risk seriously, participate actively in necessary early screenings, and proactively improve their lifestyle habits. However, this is contingent upon having sufficient data from populations with similar backgrounds to construct accurate predictive models.”*

**2. The authors used linear regressions to demonstrate that the impact of various clinical factors on BMD prediction differs across cohorts, even within the same ethnicity (Line 489-497). A more detailed exploration of the underlying reasons for these differences would be informative in the Discussion.**

Response:

Thank you for your insightful review and suggestions. We have added a section to explain the underlying reasons (Page 29-30, Line 798-808):

*“****To explore this further, we designed a straightforward experiment by developing a LR model that incorporates only clinical factors to predict BMD for each dataset. We found that that the coefficients for each clinical factor, including age and weight, exhibited considerable variability among across non-UKBB European cohorts and independent testing datasets in contrast to the training set.* *The most striking explanation for these variations is the disparate age distributions and sex ratios present in the datasets. Moreover, differences in diet, physical activity levels, sun exposure, and other lifestyle and environmental factors across populations can result in inconsistent impacts of these clinical factors on BMD, even among individuals from the similar ethnic background. These variances may represent another contributing factor to the diminished predictive capability of our model in different populations.****”*

**3. The study grouped all non-Caucasian ethnic populations within the UKBB into a into a single dataset (Line 229-241). For a more nuanced analysis, it would be advantageous todelineate these populations byspecific ethnicities. This would enhance the comparability of the model's performance with other ethnic groups in independent cohorts.**

Response:

Thank you for all the valuable reminders and suggestions. To address this issue, we have separated the East Asian and African ancestry populations from the UKBB other ancestry dataset, ultimately creating three additional ethnic datasets within the UKBB. We have made corresponding revisions in the manuscript, including the Abstract, Methods, Results, as well as the related figures and tables. All of the revisions are listed as following:

(Page 2, Line 34-40) *“We developed BMD prediction models for the femoral neck (FNK) and lumbar spine (SPN) using various methods within a UK Biobank (UKBB) training set comprising 17,964 individuals from the British white population. Models based on Regression with Least Absolute Shrinkage and Selection Operator (LASSO), selected based on the coefficient of determination (R^2^) from a model selection subset of 5,973 individuals from the British white population, underwent testing on five UKBB test sets and 12 independent cohorts of diverse ancestries, totaling over 15,000 individuals.”*

(Page 12-13, Line 338-340) *“The rest of the participants with DXA-BMD measurements in UKBB were divided to UKBB other White set (N = 1,960), UKBB African ancestry set (N = 218), UKBB East Asian ancestry set (N = 112) and UKBB other ancestry set (N = 795).”*

(Page 20, Line 530-535) *“In total, we included 320,182 individuals from UKBB for model construction and evaluation in our study, which were split to 8 data sets: the UKBB British Training set (N=17,964), the UKBB British Model Selection set (N=5,973), the UKBB British Test set (N=5,977), the UKBB other White Test set (N=1,960), the UKBB African ancestry set (N = 218), the UKBB East Asian ancestry set (N = 112), the UKBB other ancestry set (N = 795), and the UKBB Fracture case-control set (N=287,183).”*

Please also see the Table 1, Table2, Fig 1, Fig 3, S3 Table, S4 Table and S5 Fig.

**4. The inclusion of additional variables such as glucocorticoid use and rheumatoid arthritis status in the Cox proportional hazards regression analysis (Lines 359-362) should be justified. Clarifying the rationale for these choices would strengthen the methodological transparency of the study.**

Response:

Thank you for your thorough review and suggestions. We incorporated additional variables such as glucocorticoid use and rheumatoid arthritis status into the fracture prediction model, as these factors have been proven to be closely associated with osteoporotic fractures and are utilized in the commonly used fracture prediction tool, Fracture Risk Assessment Tool (FRAX). Additional content has been added to the Methods section to elucidate this point (Page 19, Line 515-518):

*“We incorporated additional variables such as glucocorticoid use and RA status into the fracture prediction model, as these factors have been proven to be closely associated with osteoporotic fractures (50, 51) and are utilized in the commonly used fracture prediction tool, Fracture Risk Assessment Tool (FRAX, https://frax.shef.ac.uk/FRAX/) (52).”*

**5. There are some detail problems in some figures and tables, such as a spelling mistake in Table 2: 'Average_DS' should be corrected to 'Average_GD'; and the full names of abbreviations like LR, PRS, etc., in Fig 2 should be clarified in the legend. A thorough review of all visual elements is recommended to ensure accuracy and clarity.**

Response:

Thank you for your thorough review; we have made the corresponding modifications. Please the Tables and Figure Legends:

a. We have corrected the spelling errors in the Table 2 and added the Legends (Page 39, Line 1107-1115):

*“FNK-BMD: Bone mineral density at femoral neck.*

*SPN-BMD: Bone mineral density at* *lumbar spine.*

*AvgGD: The average Genetic distance of the individuals in the testing set to the training set.*

*R2: The coefficient of determination.*

*PCC: Pearson correlation coefficient.*

*Lower bound: Lower bound of the 95% confidence interval.*

*Upper bound: Upper bound of the 95% confidence interval.”*

b. We have added the Legends for the Fig 2:

*“FNK-BMD: Bone mineral density at femoral neck.*

*SPN-BMD: Bone mineral density at lumbar spine.*

*R2: The coefficient of determination.*

*LR: Linear regression.*

*PRS: polygenic risk score.*

*LASSO: Regression with Least absolute shrinkage and selection operator.*

*CNN: Convolutional neural network”*

Thank you for all the insightful review and suggestions. We hope that our revisions have addressed your concerns and improved the quality of our manuscript.

-----------------------------------------------------------

**Comments from the Academic Editor:**

**I agree with offering major revision. However, I understand the reservations of the editorial team. From a clinician's perspective, I am not sure these findings will have direct clinical implications. To date, there is no routine screening of genetic factors for common diseases such as osteoporosis, since the cost-effectiveness of such procedure is unknown. It would be helpful for the authors to comment in this respect.**

Response:

Thank you for your insightful review and suggestions. We fully agree that it is challenging to quantify the economic benefits of genomic prediction models at this stage. However, we believe their clinical significance is evident. Currently, there is no large-scale population screening for common diseases such as osteoporosis. One reason for this is that such examinations (e.g., DXA scans) may be costly or lack sufficient medical resources. In this context, if clinical information and samples needed for genomic analysis (such as blood) can be collected during routine check-ups, and these models are used to preliminarily assess an individual's risk for various diseases, it could provide recommendations for further specialized examinations or clinical interventions. This approach could address personal costs while also saving public healthcare expenses. We have included a discussion on this aspect in the discussion section, including the usefulness of such models, the potential benefits and barriers to its implementation in clinical practice at current (Page Line):

We added some contents in the Discussion section, including the usefulness of such models, the potential benefits and barriers to its implementation in clinical practice at current (Page 25-26, Line 684-703):

*“Therefore, for areas where DXA measurement is inconvenient, obtaining genetic information may be more convenient and cost-effective. Moreover, building prediction models based on genetic information may be particularly useful in this situation, because the one-time cost of genotyping can be used not only for evaluation of osteoporosis but also for prediction of other complex diseases (55-58). For instance, such genetic-factor-based prediction approaches have proven effective for diseases such as atrial fibrillation, coronary artery disease, Type 2 diabetes, breast cancer, colorectal cancer, and prostate cancer (21, 58-62). Furthermore, it has been reported that genetic pre-screening could reduce the number of screening tests needed to identify individuals at risk of osteoporotic fractures, potentially enhancing the efficiency of osteoporosis screening programs (63). An additional benefit of disease risk prediction models based on genetic information is the ability to consider early identification of high-risk individuals in primary prevention strategies. It is imperative to highlight that the predictive models should serve as tools to support, not replace, the clinical judgment of physicians and the discernment of individuals. Decisions regarding clinical examinations or proactive interventions should not be made on the predictive outcomes alone. Vigilance against disease risks must be maintained, even if the model indicates a lower probability of illness. A realistic expectation for our approach is to identify individuals who have a higher risk of osteoporosis or fractures compared to the general populace, thereby encouraging them to recognize potential health threats, embrace positive lifestyle changes, and pursue timely diagnosis and preventive measures.”*

We hope that our revisions have addressed your concerns.

-----------------------------------------------------------

**General editorial requests:**

**1. Please upload any figures associated with your paper as individual TIF or EPS files with 300dpi resolution at resubmission; please read our figure guidelines for more information on our requirements: http://journals.plos.org/plosmedicine/s/figures. While revising your submission, please upload your figure files to the PACE digital diagnostic tool, https://pacev2.apexcovantage.com/. PACE helps ensure that figures meet PLOS requirements. To use PACE, you must first register as a user. Then, login and navigate to the UPLOAD tab, where you will find detailed instructions on how to use the tool. If you encounter any issues or have any questions when using PACE, please email us at PLOSMedicine@plos.org.**

**2. Please ensure that the paper adheres to the PLOS Data Availability Policy (see http://journals.plos.org/plosmedicine/s/data-availability), which requires that all data underlying the study's findings be provided in a repository or as Supporting Information. For data residing with a third party, authors are required to provide instructions with contact information for obtaining the data. PLOS journals do not allow statements supported by "data not shown" or "unpublished results." For such statements, authors must provide supporting data or cite public sources that include it.**

**3. We ask every co-author listed on the manuscript to fill in a contributing author statement, making sure to declare all competing interests. If any of the co-authors have not filled in the statement, we will remind them to do so when the paper is revised. If all statements are not completed in a timely fashion this could hold up the re-review process. If new competing interests are declared later in the revision process, this may also hold up the submission. Should there be a problem getting one of your co-authors to fill in a statement we will be in contact.** **YOU MUST NOT ADD OR REMOVE AUTHORS UNLESS YOU HAVE ALERTED THE EDITOR HANDLING THE MANUSCRIPT TO THE CHANGE AND THEY SPECIFICALLY HAVE AGREED TO IT. You can see our competing interests policy here: http://journals.plos.org/plosmedicine/s/competing-interests.**
