# Supplementary material for: Variability in performance of genetic-enhanced DXA-BMD prediction models across diverse ethnic and geographic populations: A risk prediction study
Source: PLoS Med. 2024 Aug 30;21(8):e1004451. doi: 10.1371/journal.pmed.1004451 (PMC11404845; doi:10.1371/journal.pmed.1004451)
Supplement: Attachment — Submitted filename: Response_R2.docx [file pmed.1004451.s014.docx]

Author's Response To Reviewer Comments

Dear Editor:

We are deeply grateful for your patient review and guidance on both the research content and manuscript formatting. Your suggestions have been immensely helpful for this manuscript and will significantly benefit our future research endeavors. We have revised the manuscript point-by-point according to all of the reviewer and editorial points. We have submitted a clean version of the paper as the main article file and a version with changes marked should as a marked-up manuscript. Page and line numbering in this response refers to the clean document. We would like to thank you again for your assistance. Hopefully, our revision will meet your and the reviewers' requirements.

To reviewer:

We greatly appreciate your thorough review and suggestions. We are pleased that most of our responses have met your expectations. Your recommendations have been immensely helpful for this manuscript and will significantly benefit our future research endeavors.

Please find our response below.

------------------------------------------------------------

**Requests from Editors:**

**DATA AVAILABILITY STATEMENT**

**Please note that a study author cannot be a contact for data inquiries. The Data Availability Statement (DAS) requires revision. In respect of the ‘raw whole genome array or sequencing (WGS) data’:**

**a) If the data are freely or publicly available, note this and state the location of the data: within the paper, in Supporting Information files, or in a public repository (include the DOI or accession number).**

**b) If the data are owned by a third party but freely available upon request, please note this and state the owner of the data set and contact information for data requests (web or email address). Note that a study author cannot be the contact person for the data.**

**c) If the data are not freely available, please describe briefly the ethical, legal, or contractual restriction that prevents you from sharing it. Please also include an appropriate contact (web or email address) for inquiries (again, this cannot be a study author).**

Response:

Thank you for your reminder! We have updated our DATA AVAILABILITY STATEMENT:

*“Data Availability*

*The UK Biobank data utilized in this study can be accessed through an application to the UK Biobank Resource at https://www.ukbiobank.ac.uk;*

*Data from the Osteoporotic Fractures in Men Study (MrOS, accession id: phs000373.v1.p1), the Women's Health Initiative Clinical Trial and Observational Study (WHI, accession id: phs000200.v12.p3) and the Cardiovascular Health Study study (CHS, accession id: phs000287.v7.p1) can be requested via the database of Genotypes and Phenotypes (dbGaP) at https://www.ncbi.nlm.nih.gov/gap.*

*The anonymized, lightweight dataset from the Louisiana Osteoporosis Study (LOS), the Kansas City Osteoporosis Study (KCOS), the China Osteoporosis Study (COS)) could obtained at Mendeley Data (https://data.mendeley.com/datasets, doi: 10.17632/p78t84md5h.1). The raw whole genome array or sequencing (WGS) data will also be available with a request on the Research Aging Biobank (https://agingresearchbiobank.nia.nih.gov).”*

**TITLE**

**Please revise your title according to PLOS Medicine's style. Your title must be nondeclarative and not a question. It should begin with main concept if possible. "Effect of" should be used only if causality can be inferred, i.e., for an RCT. Please place the study design ("A randomized controlled trial," "A retrospective study," "A modelling study," etc.) in the subtitle (ie, after a colon).**

Response:

Thank you for your reminder! We have updated our title as :

*“Variability in performance of genetic-enhanced DXA-BMD prediction models trained on the UK Biobank across ethnic and geographical populations: A genetic risk prediction study”*

**ABSTRACT**

**Please structure your abstract using the PLOS Medicine headings (Background, Methods and Findings, Conclusions).**

**Please combine the Methods and Findings sections into one section, “Methods and findings”.**

Response:

Thank you for your reminder! Our abstract already meets the structural requirements.

**Abstract Background: Please provide context of why the study is important. The final sentence should clearly state the study question.**

**Abstract Background: Please provide context of why the study is important. The final sentence should clearly state the study question.**

Response:

Thank you for your reminder! We added a sentence at the end of this section to clearly state the study question (Page 2, Line 31-32):

*“This study aims to develop BMD prediction models using data from the UK Biobank (UKBB) and test their performance across different ethnic and geographical populations.”*

**Abstract Methods and Findings:**

**Please ensure that all numbers presented in the abstract are present and identical to numbers presented in the main manuscript text.**

Response:

Thank you for your reminder! We have checked all numbers presented in the abstract and confirmed that they are consistent with those in the main text.

**Please include the study design, population and setting, number of participants, years during which the study took place, length of follow up, and main outcome measures.**

Response:

Thank you for your reminder! This information has been included in the Methods and Findings section (Page 2, Line 34-43).

**Please quantify the main results with 95% CIs and p values.**

Response:

Thank you for your reminder! We have updated the Abstract Methods and Findings section to present the main results with 95% confidence intervals (CIs) and p-values (Page 2-3, Line 44-54):

*“With single-nucleotide polymorphism (SNP) inclusion thresholds at* $5\times{10}^{-6}$and $5\times{10}^{-7}$*, the prediction models for FNK-BMD and SPN-BMD achieved the highest R² of 27.70% with a 95% confidence interval (CI) of [27.56%, 27.84%] and 48.28% (95% CI [48.23%, 48.34%]), respectively. Adding genetic factors improved predictions slightly, explaining an additional 2.3% variation for FNK-BMD and 3% for SPN-BMD over clinical factors alone. Survival analysis revealed that the predicted FNK-BMD and SPN-BMD were significantly associated with fragility fracture risk in the European white population (P < 0.001). The Hazard Ratios (HRs) of the predicted FNK-BMD and SPN-BMD were 0.83 (95% CI [0.79, 0.88], corresponding to a 1.44% difference in 10-year absolute risk) and 0.72 (95% CI [0. 68, 0. 76], corresponding to a 1.64% difference in 10-year absolute risk) respectively, indicating that for every increase of one standard deviation in BMD, the fracture risk will decrease by 17% and 28% respectively.”*

**Please include the important dependent variables that are adjusted for in the analyses.**

Response:

Thank you for your reminder! The important variables used in our study have been simplified described in this section (Page 2, Line 35-37):

*“We developed BMD prediction models for the femoral neck (FNK) and lumbar spine (SPN) using both genetic variants and clinical factors (such as sex, age, height, and weight), within 17,964 British white individuals from UKBB.”*

**Please include the actual amounts and/or absolute risk(s) of relevant outcomes (including NNT or NNH where appropriate), not just relative risks or correlation coefficients. (example for absolute risks: PMID: 28399126).**

Response:

Thank you for your reminder! The important variables used in our study have been simplified described in this section (Page 2-3, Line 50-54):

*“The Hazard Ratios (HRs) of the predicted FNK-BMD and SPN-BMD were 0.83 (95% CI [0.79, 0.88], corresponding to a 1.44% difference in 10-year absolute risk) and 0.72 (95% CI [0. 68, 0. 76], corresponding to a 1.64% difference in 10-year absolute risk) respectively, indicating that for every increase of one standard deviation in BMD, the fracture risk will decrease by 17% and 28% respectively.”*

**Please include a summary of adverse events if these were assessed in the study.**

Response:

Thank you for your reminder! The summary of fracture outcome has been included in the manuscript. Please see Table 1 and table 3.

**In the last sentence of the Abstract Methods and Findings section, please describe the main limitation(s) of the study's methodology.**

Response:

Thank you for your reminder! We have added a sentence to describe the main limitation(s) of the study's methodology (Page 3, Line 56-57):

*“The limitations of this study include differences in clinical factors distribution and the use of only SNPs as genetic factors.”*

**Abstract Conclusions:**

**Please address the study implications without overreaching what can be concluded from the data; the phrase "****In this study, we** **observed ..." may be useful.**

**Please interpret the study based on the results presented in the abstract, emphasizing what is new without overstating your conclusions.**

**Please avoid vague statements such as "these results have major implications for policy/clinical care". Mention only specific implications substantiated by the results.**

**Please avoid assertions of primacy ("We report for the first time....")**

Response:

Thank you for your reminder! We have updated this section (Page 3, Line 59-64):

*“In this study, we observed that combining genetic and clinical factors improves BMD prediction compared to clinical factors alone. Adjusting inclusion thresholds for genetic variants (e.g.,* $5\times{10}^{-6}$or $5\times{10}^{-7}$*) rather than solely considering genome-wide association study (GWAS)-significant variants can enhance the model’s explanatory power. The study highlights the need for training models on diverse populations to improve predictive performance across various ethnic and geographical groups.”*

Response:

In addition, we have revised the overall presentation of the abstract to reduce its length. Please refer to the whole Abstract section (Page 2-3, Line 25-64).

**STATISTICAL REPORTING**

**Previously we asked, “Please include the actual amounts and/or absolute risk(s) of relevant outcomes (including NNT or NNH where appropriate), not just relative risks or correlation coefficients. (example for absolute risks: PMID: 28399126).” We did not see any amendments related to this comment or any specific response rebuttal. Throughout, including the abstract, please report measurements of actual risk, this is a prerequisite to publication.**

Response:

Thank you for your reminder! We have added the actual amounts and absolute risks of fracture outcome at the corresponding sections of Results and Abstract:

Abstract section (Page 2-3, Line 50-54):

*“The Hazard Ratios (HRs) of the predicted FNK-BMD and SPN-BMD were 0.83 (95% CI [0.79, 0.88], corresponding to a 1.44% difference in 10-year absolute risk) and 0.72 (95% CI [0. 68, 0. 76], corresponding to a 1.64% difference in 10-year absolute risk) respectively, indicating that for every increase of one standard deviation in BMD, the fracture risk will decrease by 17% and 28% respectively.”*

Results section(Page 22, Line 591-596):

*“The HRs of the predicted FNK-BMD and SPN-BMD were 0.83 (95% CI [0.79, 0.88], corresponding to a 1.44% difference in 10-year absolute risk per standard deviation of BMD) and 0.72 (95% CI [0. 68, 0. 76], corresponding to a 1.64% difference in 10-year absolute risk per standard deviation of BMD) respectively, which means that for every increase of one standard deviation in BMD, the fracture risk will decrease by 17% and 28% respectively (Fig 5D).”*

In addition, we have added a sentence to describe how to calculate the absolute risk in Methods (Page 17, Line 459-461):

*“The hazard ratios (HRs) and 95% CIs for fracture incidence based on predicted bone mineral densities (BMDs) were estimated using a Cox model, and the absolute risk was also calculated [51].”*

**AUTHOR SUMMARY**

**Thank you for including an author summary which requires some revision. The authors summary should consist of 2-3 succinct bullet points under each of the following headings:**

**• Why Was This Study Done? Authors should reflect on what was known about the topic before the research was published and why the research was needed.**

**• What Did the Researchers Do and Find? Authors should briefly describe the study design that was used and the study’s major findings. Do include the headline numbers from the study, such as the sample size and key findings.**

**• What Do These Findings Mean? Authors should reflect on the new knowledge generated by the research and the implications for practice, research, policy, or public health. Authors should also consider how the interpretation of the study’s findings may be affected by the study limitations. In the final bullet point of ‘What Do These Findings Mean?’, please describe the main limitations of the study in non-technical language.**

**Please amend as outlined above.**

Response:

Thank you for your thorough review! We have updated the AUTHOR SUMMARY, condensing the main content into succinct bullet points (Page 4-5, Line 83-114):

*“****Author summary***

***Why was this study done?***

- *Osteoporosis diagnosis via BMD measurement (DXA) is impractical for large-scale screening, especially in resource-limited areas.*
- *Genomic data offers a cost-effective alternative for predicting disease risk, but current methods often overlook sub-significant variants and clinical factors.*
- *Most existing genomic prediction methods are based on European ancestry, with limited evaluation in other ethnic populations.*

***What did the researchers do and find?***

- *We developed BMD prediction models for femoral neck (FNK) and lumbar spine (SPN) using a training set of 17,964 individuals from British White ancestry in UK Biobank (UKBB), integrating clinical and genetic factors.*
- *We observed that strong correlations between predicted and true BMDs (R^2^≈25% for FNK-BMD and R^2^≈45% for SPN-BMD) and significant associations with fracture risk in European ancestry populations. And we identified the optimal P-value thresholds for FNK-BMD (*$5\times{10}^{-6}$*) and SPN-BMD (*$5\times{10}^{-7}$*), noting that these thresholds vary by trait and sample size.*
- *By applying the prediction models on five UKBB test sets and 12 independent cohorts of diverse ancestries, totaling over 15,000 individuals, we observed that the BMD prediction models performed well in UKBB European populations but less effectively in other ancestry groups and independent cohorts.*

***What do these findings mean?***

- *We show that genetic factors could improve the performance of DXA-BMD prediction. Within the same population, the predicted BMDs can help prioritize individuals at high risk of fragility fracture for tailored treatments.*
- *Genetic prediction methods need rigorous evaluation before application to different populations, emphasizing the importance of diverse population training.*
- *Study limitations include differences in the distribution of clinical factors such as sex and age between the UKBB datasets and the independent cohorts, as well as the inclusion of only single nucleotide polymorphisms (SNPs) as genetic factors.”*

**INTRODUCTION**

**Please conclude the Introduction with a clear description of the study question or hypothesis.**

Response:

Thank you for your reminder! We have updated the final paragraph of the Introduction to include a description of the research question (Page 7-8, Line 197-206):

*“Our study aims to address these gaps by developing prediction models that integrate genetic and clinical factors, for DXA-BMD at two clinically relevant sites: the femoral neck (FNK) and lumbar spine (SPN). These sites are not only pivotal for osteoporosis diagnosis but also serve as the most significant predictors of fracture risk [22]. By leveraging a larger cohort and incorporating a broader array of genetic variants, our model seeks to provide a more accurate and comprehensive reflection of BMD variation, thereby enhancing the predictive precision for osteoporosis and associated fracture risks. Further, to investigate whether the prediction models developed in the UKBB European population could be applied to other groups, we tested our models in multiple cohorts of diverse ancestry and geographic backgrounds across populations.”*

**METHODS and RESULTS**

**Please report the number of [patients, samples, etc] and dates of recruitment, and account for all methods used in your study.**

Response:

Thank you for your reminder! These details have already been included in the manuscript.

**Please define "lost to follow-up" as used in this study. Other reasons for exclusion should be defined.**

Response:

Thank you for your reminder! In our study, we used electronic health records to obtain fracture outcomes for individuals. We did not include individuals lacking electronic health records, so there was no need to define “lost to follow-up.” We have added a note about this in the Methods section (Page 12, Line 330-331):

*“Individuals without any hospital inpatient data were excluded from this study.”*

**Please define the length of follow up (eg, in mean, SD, and range).**

Response:

Thank you for your reminder! For the UKBB fracture case-control dataset, we obtained the incidence of fractures within 10 years after the initial assessment. The length of follow up was defined as the time from the initial assessment to the first occurrence of a fragility fracture or the individual’s death (up to a maximum of 10 years). We separately calculated the length of follow-up for the Case and Control groups and added this information to the corresponding section of the S1 Appendix (Page 5, Line 91-99):

*“The individuals with any fragility fracture code within the next 10 years were defined as the fragility fracture cases, and their follow-up time to fracture occurrence was also captured. The follow-up duration was defined as the time from the initial assessment to the first occurrence of a fragility fracture or the individual’s death, up to a maximum of 10 years. In total, we identified 17,490 fragility fractures cases using ICD10 and self-reported codes, and 269,693 individuals as controls. For the Case group, the average follow-up duration was 6.54 years with a standard deviation of 3.20 years. For the Control group, the average follow-up duration was 9.80 years with a standard deviation of 1.04 years.”*

**Please provide the actual numbers of events for the outcomes, not just summary statistics or ORs.**

Response:

Thank you for your reminder! We have added the actual numbers of events for the fracture outcome. Please refer to Table 3.

**Please present numerators and denominators used to derive percentages.**

Response:

Thank you for your reminder! We have updated a section in the Results for this (Page 21, Line 565-571):

*“For example, among individuals with a predicted FNK-BMD below the 5th percentile, 1,713 out of 14,360 (11.93%) experienced a fragility fracture within the next 10 years. Similarly, among those with a predicted SPN-BMD below the 5th percentile, 1,557 out of 14,360 (10.84%) experienced a fragility fracture. In contrast, the prevalence of fragility fractures was significantly lower among individuals with a predicted BMD above the 95th percentile, with only 3.84% (551 out of 14,360) for FNK-BMD and 3.93% (564 out of 14,360) for SPN-BMD.”*

**Please ensure to indicate where analyses are adjusted and which factors are adjusted for.**

Response:

Thank you for your reminder! The adjusted analyses and the factors considered have been detailed in the Methods section (Page 17, Line 453-456):

*“Then, we performed a multivariate survival analysis with the Cox proportional hazards regression (Cox) model, integrating the predicted BMDs and clinical factors: age, sex, height, weight, body mass index (BMI), previous fracture, smoking, drinking, exercise, glucocorticoid using and RA.”*

**As for the abstract, please ensure to quantify the main results with 95% CIs and p values.**

Response:

Thank you for your reminder! We have updated the Abstract Methods and Findings section to present the main results with 95% confidence intervals (CIs) and p-values.

**When a p value is given, please specify the statistical test used to determine it.**

Response:

Thank you for your reminder! All the statistical test used in this study have been specified.

**The methods section is very long and it may help to improve reader accessibility if some of the details could be moved to the supporting information files.**

Response:

Thank you for your reminder! We have moved some of the details in the “Study cohorts and data preprocessing”, “Phenotype measurements and quality control” and “Prediction models of BMDs” to the supporting information files. Please refer to the Methods section and S1 Appendix.

**Line 242 – Study cohorts and data preprocessing – this section is particularly long and contains information regarding datasets and statistical methods. Suggest separating this information into two different sections (databases/cohorts and statistical methods) to improve reader accessibility.**

Response:

Thank you for your reminder! We have moved some of the details to the supporting information files. And we separated this part into two sections: “Study cohorts” and “Data preprocessing” (Page 9-11, Line 249-303):

*“****Study cohorts***

*We obtained the data sets for our study from 7 studies: the UKBB Study [23], the Louisiana Osteoporosis Study (LOS) [24], the Kansas City Osteoporosis Study (KCOS), the China Osteoporosis Study (COS), the Osteoporotic Fractures in Men Study (MrOS) [25], the Women's Health Initiative Clinical Trial and Observational Study (WHI) [26] and the Cardiovascular Health Study (CHS) [27]:*

*The UKBB is a large-scale biomedical database and research resource containing genetic, lifestyle and health information from more than 500,000 UK participants, enrolled at ages from 40 to 69 [28]. In total, 32,999 independent participants with DXA-BMD measurements in UKBB were used for model training and evaluation in our study. To validate the association between predicted BMDs and fragility fracture risk, we applied our prediction approaches and evaluated the association between the predicted BMDs and incidence of fragility fracture in 287,183 participants (17,490 fragility fracture cases and 269,693 controls) without DXA-BMD in UKBB. The current research project (application number 63047) was approved by UKBB.*

*The LOS study is an ongoing cross-sectional study with more than 17,000 subjects so far since 2011 for investigating genetic and nongenetic determinants of osteoporosis and other complex diseases/traits. In total, we included 2,863 Caucasians and 2,097 African Americans randomly selected (stratified by sex and race groups) from the whole LOS cohort[24]. We separated the cohort into LOS African Americans (LOS_AFR) set and LOS Caucasians (LOS_CAU) set when evaluating the performance of prediction models.*

*The KCOS study included 2,271 unrelated subjects of European ancestry from the Kansas City osteoporosis study (KCOS_CAU). The COS study included 1,569 unrelated subjects of East Asian (Chinese Han) ancestry from the China osteoporosis study (COS_EAS). More details about the sample processing, DNA extraction as well as variant calling can be found in previous published studies [29].*

*The MrOS is an international multi-center longitudinal study of elderly men. The MrOS cohort recruited 5,995 men aged ≥ 65 years at multiple assessment centers in the USA between 2000 and 2002 [25]. Among them, we included participants with both genotype data and DXA-BMD measurements. Subsequently, we segregated them based on ancestry, creating four distinct data sets: 4,587 individuals of Caucasian ancestry (MrOS_CAU), 181 individuals of African American ancestry (MrOS_AFR), 165 individuals of East Asian ancestry (MrOS_EAS) and 111 individuals of Hispanic ancestry (MrOS_HIS).*

*The WHI study is a long-term national health investigation that has focused on strategies for preventing heart disease, breast and colorectal cancer, as well as osteoporotic fractures in postmenopausal women [26]. In this study, we included 1,064 individuals with both genotype data and DXA-BMD measurements. Subsequently, we segregated them by ancestry: 671 individuals of Black or African-American ancestry (WHI_AFR) and 393 individuals of Hispanic/Latino ancestry (WHI_HIS).*

*The CHS is a prospective investigation aimed at identifying risk factors for the development and progression of coronary heart disease and stroke in individuals aged 65 years and older [27]. We categorized the CHS cohort by ancestry into two sets: 432 individuals of White/Caucasian ancestry (CHS_CAU) and 190 individuals of Black/African-American ancestry (CHS_AFR).*

*All samples were approved by the respective institutional ethics review boards, and all participants provided written informed consent.*

***Data preprocessing***

*For UKBB cohort, we extracted the participants who underwent the DXA measurement to develop the BMD prediction models. Considering the requirement for a substantial number of samples for the model training and promotion, we have adopted a relatively stringent criterion for the exclusion of outlier samples. Specifically, we exclude samples with extreme BMD values below or above the 0.1th percentile to mitigate potential errors that may arise from handing factors. To ensure the independence of our samples, the kinship between each pair of individuals was inferred using KING software [30]. We randomly retained only one individual from the inferred kinship relationships within 3rd-degree or closer, while the others were excluded. All genotype data obtained using chip-based genotyping were imputed using the Haplotype Reference Consortium (HRC), the UK10K, or the 1000G reference panel [31, 32]. For more detailed data preprocessing procedures, please refer to S1 Appendix.”*

**Line 529 – suggest ‘****Characteristics of the study population’ as an alternative sub-heading.**

Response:

Thank you for your suggestion! We have revised the subheading of this section to ‘Characteristics of the study population’ (Page 18, Line 472).

**TABLES and FIGURES**

**Please see here for guidelines on submitting and citing figures https://journals.plos.org/plosmedicine/s/figures#loc-how-to-submit-figures-and-captions**

**Please provide titles and legends for all tables and figures (including those in Supporting Information files).**

Response:

Thank you for your reminder! We have checked all the titles and legends for all tables and figures (including those in Supporting Information files).

**Please ensure that each table and figure is affiliated to a caption which clearly describes the figure content without the need to refer to the text.**

Response:

Thank you for your reminder! We have reviewed all the charts to ensure that each table and figure has an accompanying caption that clearly explains the content without the need to refer to the main text.

**Please ensure that all abbreviations are defined in the caption or an appropriate footnote, including those used to report statistical information.**

Response:

Thank you for your reminder! We have check reviewed all the Figure and Tables, and all the abbreviations are defined in the caption or an appropriate footnote.

**Please ensure to include the meaning of any dots/lines/bars.**

Response:

Thank you for your reminder! We have check reviewed all the Figure and Tables, and the meaning of any dots/lines/bars is indicated in the corresponding legends.

**Please consider avoiding the use of red and green in order to make figures more accessible to those with colour blindness.**

Response:

Thank you for your reminder! We have updated all the color figures in the main text. to ensure that red and green are not used to mark groups.

**To help facilitate transparent data reporting, where adjusted analyses are presented please also present the unadjusted analyses for comparison. In a caption or footnote please detail all factors adjusted for.**

Response:

Thank you for your reminder! Our study did not include adjusted analyses.

**DISCUSSION**

**Previously we asked, ‘Please present and organize the Discussion as follows: a short, clear summary of the article's findings; what the study adds to** **existing research and where and why the results may differ from previous research; strengths and limitations of the study; implications and next steps for research, clinical practice, and/or public policy; one-paragraph conclusion.’**

**Reading through, the discussion is very long and it is easy to get lost. Please revise for brevity and improve accessibility ensuring that the above structure is followed and the items easily identifiable. Pleas avoid the use of subheadings.**

Response:

Thank you for your thorough review and reminder! We have simplified and reorganized the Discussion section. Please refer to the Discussion section (Page 25-29, Line 655-789).

**REFERENCES**

**For in-text reference callouts please place citations in square parentheses separate by commas. For example, [1,3,6] or [1-3]. Please check and amend throughout all sub-sections of the manuscript and supporting files.**

**In the bibliography please ensure that you list up to but no more than 6 author names followed by et al.**

**For all web references please ensure you include an, ‘Accessed [date].’**

**Journal name abbreviations should be those listed in the National Center for Biotechnology Information (NCBI) databases.**

Response:

Thank you for your reminder! Due to the simplification and update of the Methods and Discussion sections, some references have been removed from the main text. Additionally, we have added a reference related to the calculation of absolute risk. Please see below for all changes related to References:

Removed references:

1. Smigielski EM, Sirotkin K, Ward M, Sherry ST. dbSNP: a database of single nucleotide polymorphisms. Nucleic Acids Res. 2000;28(1):352-5.
2. Srivastava N, Hinton G, Krizhevsky A, Sutskever I, Salakhutdinov R. Dropout: a simple way to prevent neural networks from overfitting. J Mach Learn Res. 2014;15(1):1929–58.
3. Goodwin S, McPherson JD, McCombie WR. Coming of age: ten years of next-generation sequencing technologies. Nat Rev Genet. 2016;17(6):333-51.
4. Inouye M, Abraham G, Nelson CP, Wood AM, Sweeting MJ, Dudbridge F, et al. Genomic Risk Prediction of Coronary Artery Disease in 480,000 Adults: Implications for Primary Prevention. J Am Coll Cardiol. 2018;72(16):1883-93.
5. Lourida I, Hannon E, Littlejohns TJ, Langa KM, Hypponen E, Kuzma E, et al. Association of Lifestyle and Genetic Risk With Incidence of Dementia. JAMA. 2019;322(5):430-7.
6. Rutten-Jacobs LC, Larsson SC, Malik R, Rannikmae K, consortium M, International Stroke Genetics C, et al. Genetic risk, incident stroke, and the benefits of adhering to a healthy lifestyle: cohort study of 306 473 UK Biobank participants. BMJ. 2018;363:k4168.
7. Forgetta V, Keller-Baruch J, Forest M, Durand A, Bhatnagar S, Kemp JP, et al. Development of a polygenic risk score to improve screening for fracture risk: A genetic risk prediction study. PLoS Med. 2020;17(7):e1003152.
8. El Miedany Y. Best Practice Recommendations for DXA Scans and Reports. In: El Miedany Y, editor. New Horizons in Osteoporosis Management. Cham: Springer International Publishing; 2022. p. 297-326.
9. Li YM, Peng C, Zhang JG, Zhu W, Xu C, Lin Y, et al. Genetic risk factors identified in populations of European descent do not improve the prediction of osteoporotic fracture and bone mineral density in Chinese populations. Sci Rep. 2019;9(1):6086.
10. Wang Y, Zhu M, Ma H, Shen H. Polygenic risk scores: the future of cancer risk prediction, screening, and precision prevention. Med Rev (2021). 2021;1(2):129-49.

Added references:

1. Du H, Li L, Bennett D, Guo Y, Turnbull I, Yang L, et al. Fresh fruit consumption in relation to incident diabetes and diabetic vascular complications: A 7-y prospective study of 0.5 million Chinese adults. PLoS Med. 2017;14(4):e1002279.

Note, these changes are solely due to the changes in the main text content.

**Line 881 – please remove the data availability statement from the main manuscript and include only in the manuscript submission form when you resubmit the manuscript. It will be compiled as metadata at the time of publication.**

Response:

Thank you for your reminder! We have moved this section from the main manuscript and submitted it through the manuscript submission system.

**SUPPORTING INFORMATION**

**In the published article, supporting information files are accessed only through a hyperlink attached to the captions. For this reason, you must list captions at the end of your manuscript file. You may include a caption within the supporting information file itself, as long as that caption is also provided in the manuscript file. Do not submit a separate caption file.**

**As the supporting information files are contained with a single file:**

**Please label the file as ‘S1 Supporting Information’.**

**Please apply alphabetical labelling to each table and figure contained within the S1 file. For example, ‘Fig A’ to ‘Fig Z’ and ‘Table A’ to ‘Table Z’.**

**Plain text does not need to be labelled and can just be given a title as necessary. For example, ‘Statistical Analysis Plan’.**

**Please cite tables/figures as ‘Fig A in S1 Supporting Information’ and/or ‘Table A in S1 Supporting Information’, for example.**

**Please cite plain text as, ‘Statistical Analysis Plan in S1 Supporting Information’, for example.**

**Alternatively, you may upload each file in the supporting information as individual files and label tables as ‘S1 Table’ (so on) and figures as ‘S1 Fig’ (and so on).**

**Any additional documents (protocols or appendices) can be labelled as ‘S1 Appendix’, for example.**

**Please cite items as exactly as labelled.**

**Further guidance can be found here https://journals.plos.org/plosmedicine/s/supporting-information**

Response:

Thank you for your reminder! We have checked all of the supporting information files and uploaded them separately.

**SOCIAL MEDIA**

**To help us extend the reach of your research, please detail any X (formerly Twitter) handles you wish to be included when we tweet this paper (including your own, your coauthors’, your institution, funder, or lab) in the manuscript submission form when you re-submit the manuscript.**

Response:

Thank you for your suggestion! But we don’t have a X account now.

**Comments from Reviewers:**

**Reviewer #1: Comments have been addressed**

**Perhaps some additional information can be provided regarding the variant rs554533790 (which genes it is close to) - if at all possible.**

Response:

Thank you for your insightful review and suggestions. The variant rs554533790 is located in the intergenic region, between *CLCA1* and *CLCA4* genes. We searched for potential functional annotations of rs554533790 using the following resources: SNIPA (https://snipa.org/snipa3), SNP-nexus (https://www.snp-nexus.org/v4), UCSC Genome Browser (https://genome.ucsc.edu), GWAS Catalog (https://www.ebi.ac.uk/gwas), and GTEx (https://www.gtexportal.org). However, we did not find any known functions for rs554533790, nor could we determine if rs554533790 has any functional relationship with these two genes. The role of this variant may need to be explored in future functional or mechanistic studies. We have added a sentence to discuss it (Page 27, Line 718-720):

*“However, as a rare variant in an intergenic region, the function of rs554533790 remains largely unknown. Further functional or mechanistic studies are needed to elucidate its role in bone.”*

**Reviewer #2: We thank the authors for largely addressing our previous concerns. On the point where one individual from inferred kinship relationships being retained, it might be detailed as to how the retained individual was selected - was it essentially random (i.e. first encountered individual retained, then any of their relations excluded)?**

Thank you for your insightful review and suggestions. Yes, the process of excluding individuals from inferred kinship relationships is essentially random. The kinship between each pair of individuals was inferred. If the pair was inferred to be within the 3rd degree, we retained the first individual and removed the second individual. We have updated the description about this in the manuscript. Please refer to the S1 Appendix (Page 1, Line 17-20):

*“To ensure the independence of our samples, the kinship between each pair of individuals was inferred. We randomly retained only one individual from the inferred kinship relationships within 3rd-degree or closer, while the others were excluded.”*

**Reviewer #3: The authors have addressed all of my comments. Thanks!**

Thank you for your insightful review and suggestions.
